# Behavioral and neurochemical interactions of the tricyclic antidepressant drug desipramine with L-DOPA in 6-OHDA-lesioned rats. Implications for motor and psychiatric functions in Parkinson’s disease

**DOI:** 10.1007/s00213-022-06238-x

**Published:** 2022-09-30

**Authors:** Kinga Kamińska, Tomasz Lenda, Jolanta Konieczny, Elżbieta Lorenc-Koci

**Affiliations:** Department of Neuro-Psychopharmacology, Maj Institute of Pharmacology, Polish Academy of Sciences, Smętna street 12, 31-343 Kraków, Poland

**Keywords:** Contralateral rotations, Desipramine, Depressive-like behavior, L-DOPA, Monoamine levels, Unilateral 6-OHDA lesion

## Abstract

**Rationale:**

The pharmacological effects of antidepressants in modulating noradrenergic transmission as compared to serotonergic transmission in a rat model of Parkinson’s disease under chronic L-DOPA therapy are insufficiently explored.

**Objectives:**

The aim of the present study was to investigate the effect of the tricyclic antidepressant desipramine administered chronically alone or jointly with L-DOPA, on motor behavior and monoamine metabolism in selected brain structures of rats with the unilateral 6-OHDA lesion.

**Methods:**

The antiparkinsonian activities of L-DOPA and desipramine were assessed behaviorally using a rotation test and biochemically based on changes in the tissue concentrations of noradrenaline, dopamine and serotonin and their metabolites, evaluated separately for the ipsi- and contralateral motor (striatum, substantia nigra) and limbic (prefrontal cortex, hippocampus) structures of rat brain by HPLC method.

**Results:**

Desipramine administered alone did not induce rotational behavior, but in combination with L-DOPA, it increased the number of contralateral rotations more strongly than L-DOPA alone. Both L-DOPA and desipramine + L-DOPA significantly increased DA levels in the ipsilateral striatum, substantia nigra, prefrontal cortex and the ipsi- and contralateral hippocampus. The combined treatment also significantly increased noradrenaline content in the ipsi- and contralateral striatum, while L-DOPA alone decreased serotonin level on both sides of the hippocampus.

**Conclusions:**

The performed analysis of the level of monoamines and their metabolites in the selected brain structures suggests that co-modulation of noradrenergic and dopaminergic transmission in Parkinson’s disease by the combined therapy with desipramine + L-DOPA may have some positive implications for motor and psychiatric functions but further research is needed to exclude potential negative effects.

**Supplementary Information:**

The online version contains supplementary material available at 10.1007/s00213-022-06238-x.

## Introduction

Parkinson’s disease (PD) is a progressive neurodegenerative disorder that affects almost 4 million people all over the word (Dorsey et al. 2007), although it is likely that this number is underestimated (Van Den Eeden et al. 2003). Clinically, the disease is defined by major motor symptoms that include slowness or absence of voluntary movements, rigidity of the limbs, resting tremor, postural instability and freezing (Jankovic 2008). The most characteristic pathological feature of the disease is the pronounced loss of dopaminergic neurons in the substantia nigra pars compacta (SNc), resulting in a drastic decline in the dopamine (DA) levels in the striatum (STR) to which these neurons project (Bernheimer et al. 1973; Kish et al. 2008). In addition to the loss of the dopaminergic nigrostriatal pathway, progressive neurodegeneration also involves the ascending mesocortico-limbic dopaminergic pathways from ventral tegmental area (VTA) (Paulus and Jellinger 1991; Ehringer and Hornykiewicz 1998), noradrenergic pathways from the locus coeruleus (LC; Chan-Palay and Asan 1989; Del Tredici et al 2002) and serotonergic pathways from the raphe nuclei (RN; Bernheimer et al. 1961; Halliday et al. 1990a, b). Degeneration of noradrenergic and serotonergic neurons in the LC and RN, respectively, precedes the loss of dopaminergic neurons in the SNc and VTA (Braak et al. 2003, 2004; Zarow et al. 2003; Del Tredici and Braak 2012, 2013; Giguère et al. 2018).

In PD, motor symptoms are mainly attributed to the loss of dopaminergic neurons in the SNc and the resulting depletion of striatal DA. On the other hand, a wide spectrum of neuropsychiatric disorders, such as depression, apathy, anxiety and mild cognitive impairment (Jankovic 2008; Chaudhuri et al. 2006; Chaudhuri and Schapira 2009), which occur in the non-motor phase of the disease and persist in its advanced stage, is primarily associated with dysfunctions of noradrenergic, serotonergic and cholinergic neuronal systems in various regions of the brain (Halliday et al. 1990a, b, Braak et al. 2003, 2004, Brichta et al. 2013, Moghaddam et al. 2017). However, in the search for the causes of mental disorders in PD, one cannot ignore the progressive impairment of dopaminergic transmission due to the degeneration of the ascending dopaminergic pathways from the VTA. Interestingly, the dopaminergic and noradrenergic projections from the VTA and LC, respectively, converge in the prefrontal cortex (PFC), where, according to more recent studies, they play a key role in the cognitive and motivational functions (Xing et al. 2016; Berridge and Spencer 2016). Thus, degeneration of these pathways may significantly contribute to the development of psychiatric symptoms in PD.

Hence, although PD has long been considered a purely motor disorder, the heterogeneous nature of neurodegeneration and the significant predominance of non-motor symptoms now allow it to be classified as a multi-system disorder (Simuni and Sethi 2008). In line with this view, studies using 6-OHDA model have revealed that, in addition to DA, also deficiency of noradrenaline (NA) and serotonin (5-HT) contributes to dysregulation of the basal ganglia (Delaville et al. 2012a, b, c) responsible for motor functions. Moreover, since both noradrenergic, dopaminergic and serotonergic transmissions in the PFC are altered in several psychiatric and neurological disorders (Hensler et al. 2013), it is almost certain that the deficiency of these neurotransmitters observed in the PFC of PD patients (Scatton et al. 1983) contributes to psychiatric disturbances in this disorder.

In light of the above data, it becomes clear that DA replacement therapy using its precursor L-DOPA is insufficient to alleviate wide range of symptoms in PD. Therefore, a new pharmacological treatment strategy for this disease must be optimized by targeting disturbances in noradrenergic and serotonergic transmissions, while supplementing DA deficiency, in order to improve motor and psychiatric symptoms (Fornai et al. 2007; Lewitt 2012; Espay et al. 2014; Lanza and Bishop 2018; Muñoz et al. 2020; Paredes-Rodriguez et al. 2020; Conti et al. 2018). Recently, an increasing body of experimental data from animal PD models confirms the beneficial effect of modulating the serotonergic transmission in the presence of L-DOPA on DA release and, consequently, on motor behavior including motor complications, such as abnormal involuntary movements (AIMs) known as dyskinesia (Bishop et al. 2012, Conti et al. 2014, 2016a, b, Fidalgo et al. 2015, De Deurwaerdère et al. 2017, Kamińska et al. 2018, Miguelez et al. 2017, Chagraoui et al. 2020). However, studies on the effect of modulation of noradrenergic transmission in the presence of L-DOPA on motor impairments are less numerous and less consistent because both improvement (Lundblad et al. 2002; Dekundy et al. 2007; Buck et al. 2010; Shin et al. 2014, Wang et al. 2014) and deterioration of the motor functions have been described in such conditions in PD models (Chotibut et al. 2012, Chotibut et al. 2014, Conti et al. 2016a, b). The beneficial effect of modulating the noradrenergic transmission in the relief of L-DOPA induced dyskinesias was mainly achieved with the use of noradrenargic drugs that are α2 adrenergic receptor antagonists, such as yohimbine or idazoxane (Lundblad et al. 2002; Dekundy et al. 2007; Buck et al. 2010, Wang et al. 2014). In contrast, modulating the noradrenergic transmission in the presence of L-DOPA by means of NA reuptake inhibitors, such as nisoxetine or desipramine (DES), resulted in worsening of dyskinesias in these models (Chotibut et al. 2014; Conti et al. 2016a, b). The elucidation of the reasons for the unfavorable effect of NA reuptake inhibitors in combination with L-DOPA on motor complications is of great therapeutic importance, especially because antidepressants modulating mainly noradrenergic transmission can be used in the treatment of depression accompanying PD, even in the presymptomatic phase of this disease. It is worth mentioning that the noradrenergic projections from the LC control both dopaminergic and serotonergic transmission and are the first to degenerate in PD (Paulus and Jellinger 1991; Braak et al. 2003, 2004). Furthermore, there is convincing evidence that NA plays a trophic and neuroprotective role (Rommelfanger and Weinshenker 2007) in various brain regions thereby limiting neurodegenerative processes in cognitive and motor circuits and promoting neurogenesis in the hippocampus (Jhang et al. 2014; Coradazzi et al. 2016; Feinstein et al. 2016; Zhu et al. 2019). The experimental data cited above clearly indicate the need to continue research on the modulation of noradrenergic transmission in the conditions of L-DOPA therapy due to the superior role of NA in controlling the functions of monoaminergic systems and its unique trophic and neuroprotective properties.

Among the tricyclic antidepressants, DES has the highest potency in inhibiting NA reuptake (IC_50_ = 0.83 nM) with relatively low potency in inhibiting 5-HT reuptake (IC_50_ = 200 nM), and is a weak antagonist at α1 adrenoreceptor (IC_50_ = 260 nM) and 5-HT_2A_ serotonin receptor (IC_50_ = 560 nM) (Hyttel 1994). Therefore, in this study, we decided to investigate how the combined therapy with DES and the commonly used antiparkinsonian drug L-DOPA affect motor behavior and tissue concentrations of NA, DA and 5-HT in selected brain structures of rats with a unilateral lesion induced by 6-OHDA. The previously developed rat 6-OHDA model of PD (Kamińska et al. 2017) was used to reflect the changes in the monoamine levels observed in the advanced stage of this disease, and to check the effectiveness of chronic administration of the tested drugs (DES, L-DOPA) in modulating these changes. In this model, 6-OHDA was administered at a dose of 16 μg/4 μl unilaterally into the MFB without prior administration of DES which is often used in order to protect noradrenergic terminals. Such method of 6-OHDA administration resulted in a drastic loss of both DA and NA contents in the STR, hippocampus (HIP) and prefrontal cortex (PFC) on the ipsilateral side, and an approximately 50% reduction in 5-HT concentration in these structures on this side. In the ipsilateral SN, only a drastic decrease in DA content was observed, while the content of NA and 5-HT remained unchanged (Kamińska et al. 2017). DES was administered at a dose of 10 mg/kg, which is used to reduce depressive-like behavior in rodents (Xie et al. 2017), while L-DOPA was injected at a dose of 12 mg/kg which is most effective in increasing extracellular DA levels both in the STR and SN (Navailles et al. 2010). Both drugs were given chronically, alone or in combination for 3 weeks. The effect of DES on motor functions, measured as L-DOPA-induced contralateral rotations, was examined in the context of improving locomotor activity based on the analysis of the tissue concentrations of NA, DA and 5-HT in rat brain motor structures (STR, SN). In our previous study (Kamińska et al. 2017), we have demonstrated that administration of a single dose of 6-OHDA (16 μg/4 ml) unilaterally into the MFB caused anhedonia (a core symptom of depression) assessed 3 weeks after the surgery as a decrease in the intake of 3% sucrose solution. However, this test is not reliable for assessing the interaction of DES and L-DOPA at the peak of L-DOPA action because the rotational behavior interferes with the consumption of sucrose solution; hence, the analysis of this interaction in the “phase on” with regard to depressive symptoms was performed only on the basis of changes in the tissue concentrations of monoamines (NA, DA and 5-HT) in the limbic structures (PFC, HIP) of the rat brain. We hope that these experiments will allow us to better assess the efficacy of the combined DES + L-DOPA therapy for both motor and psychiatric symptoms.

## Materials and methods

The experiments were performed in accordance with the Act on the Protection of Animals Used for Scientific or Educational Purposes of January 21, 2005 reapproved on January 15, 2015 (published in Journal of Laws no 23/2015 item 266, Poland), and in compliance with the Directive of the European Parliament and of the Council of Europe 2010/63/EU of 22 September 2010 on the protection of laboratory animals. The experimental protocols were approved by the Ethics Committee at the Institute of Pharmacology, Polish Academy of Sciences, Krakow, Poland (permission no 709/2010 of 28 January 2010). During the experiments, efforts were made to minimize the suffering of animals, and their numbers were limited to the minimum necessary to obtain statistically reliable results.

### Animals

Male Wistar Han rats (Charles River, Sulzfeld, Germany) with an initial body weight between 290 and 320 g were housed in standard plastic rodent cages (5 animals per cage). Animals were kept under standard laboratory conditions, i.e. at a room temperature of 22 ± 2 °C, 45–65% humidity and a 12-h light/dark cycle. Standard laboratory rodent food and water were available ad libitum. The animals were experimentally naive.

### Drugs

Benserazide hydrochloride, desipramine hydrochloride (DES), 3,4-dihydroxy-L-phenylalanine methyl ester (L-DOPA), 6-hydroxydopamine hydrochloride (6-OHDA), L-ascorbic acid, R-( −)-apomorphine hydrochloride hemihydrate were provided by the Sigma-Aldrich Chemical Company (Steinheim, Germany). If not stated otherwise all other compounds were provided by the Sigma-Aldrich Chemical Company (Steinheim, Germany).

### Experimental design

**Fig. 1:**
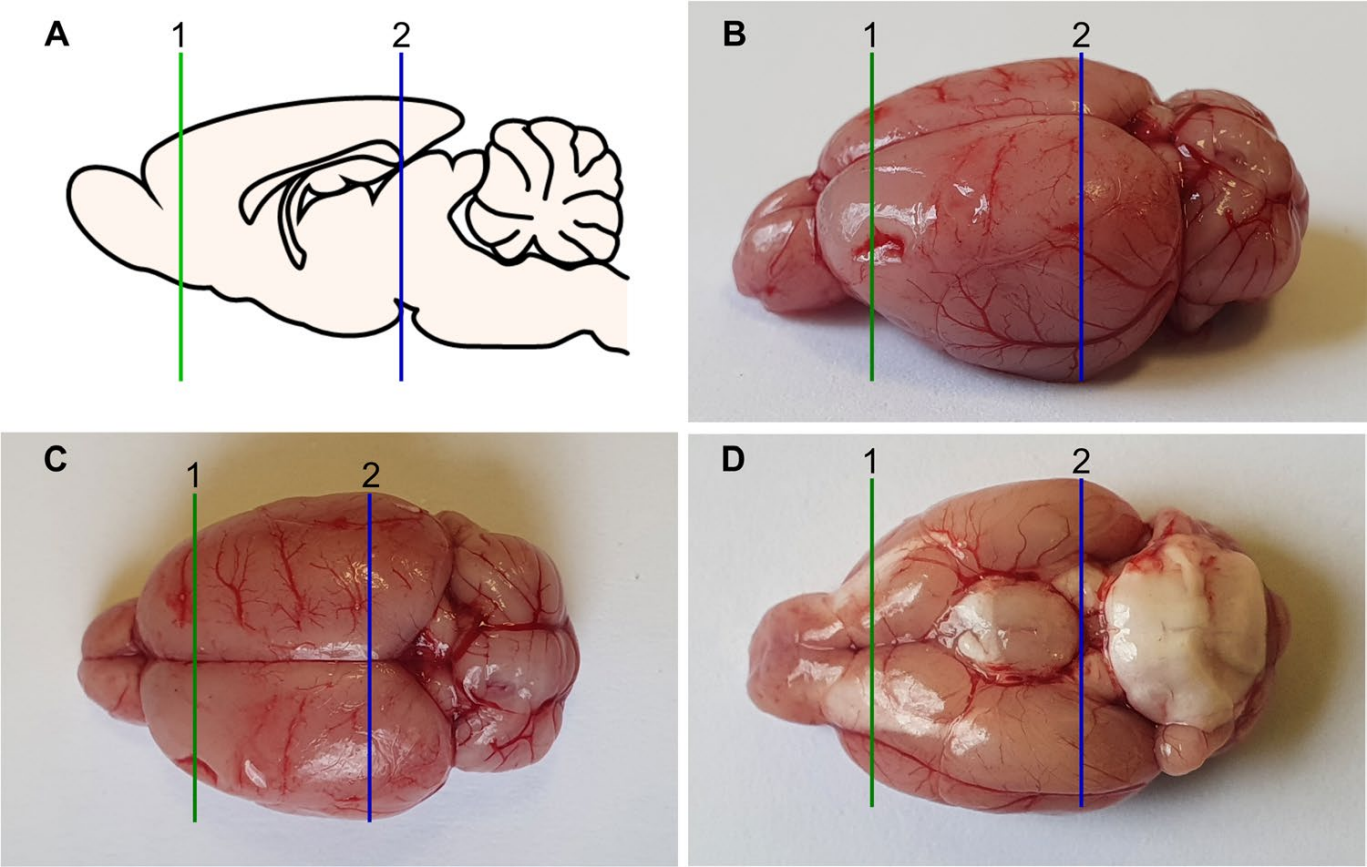
Dissection of an adult rat brain—lateral (**A**, **B**), dorsal (**C**) and ventral (**D**) view. From the isolated brains, the ipsi- and contralateral sides of the motor (STR, SN) and limbic (PFC, HIP) structures were separately dissected, immediately frozen on dry ice and stored at − 80 °C. The lines show the placement of transverse cuts: the first (1) and the second (2) one

To examine the interaction of DES with L-DOPA, we used our previously developed rat model of symptomatic PD based on unilateral 6-OHDA injection, with coexisting depressive-type behavior measured as anhedonia (Kamińska et al. 2017). On day 13^th^ after 6-OHDA administration into the left medial forebrain bundle (MFB) rats were tested for the rotational behavior induced by apomorphine (0.25 mg/kg s.c.) to determine the extent of the lesion. Our earlier biochemical and histological studies (Lorenc-Koci et al. 2013; Czarnecka et al. 2013; Kamińska et al. 2017) confirmed that rats exhibiting more than 100 contralateral turns/1 h, developed the extensive unilateral lesion of the nigrostriatal dopaminergic system. Therefore, only rats with a total number of rotations of 100 or more per 1 h were selected for further behavioral and biochemical studies. These rats were randomly assigned to equally rotating groups, and, starting on the day following the apomorphine test, they were administered solvent, DES (10 mg/kg ip), L-DOPA (12 mg/kg ip) or the DES + L-DOPA combination once daily for 21 consecutive days. Benserazide hydrochloride (6.25 mg/kg i.p.), an inhibitor of peripheral aromatic acid decarboxylase (AADC), was injected 30 min while DES 10 min before L-DOPA. Benserazide hydrochloride and DES were dissolved in redistilled water, L-DOPA methyl ester in 0.9% NaCl and all of these drugs were given in a volume of 1 ml/kg. Throughout the text, the combined administration of benserazide hydrochloride + L-DOPA methyl ester is referred to as L-DOPA treatment. Rotational behavior was recorded for 120 min immediately after the first and the penultimate doses of the examined drugs. On the next day after the second rotation test, rats were given the last drug doses, and 60 min later they were sacrificed by decapitation. From the isolated brains, the ipsi- and contralateral sides of the motor (STR, SN) and limbic (PFC, HIP) structures were separately dissected immediately frozen on dry ice and stored at − 80 °C until further procedures were applied. More information on the procedure of cutting out selected brain structures is presented in Fig. 1. This figure shows a diagram of a sagittal cross section of the rat brain and photos of the brain from the lateral, dorsal and ventral sides; in addition, the locations of subsequent transverse cuts allowing access to the brain structures that were intended for dissection are marked, separately from the right and left hemisphere.

### Stereotaxic surgery

Stereotaxic brain surgery was performed under ketamine (50 mg/kg, Biowet, Poland) and diazepam (2.5 mg/kg, Polfa Warszawa, Poland) anesthesia. Both drugs were administered i.p., as a mixture (1:1 v/v) in a volume of 1 ml/kg of body weight, and then each anesthetized rat was placed in a stereotaxic apparatus (David Kopf Instruments, Tujunga, CA, USA). 6-OHDA at a single dose of 16 μg (calculated as the free base) was given via a stainless steel needle (0.28 mm o.d.) inserted unilaterally through a small hole in the skull directly into the left MFB as described previously (Kamińska et al. 2017, 2018). The stereotaxic coordinates determined according to the atlas of Paxinos and Watson (2007) were as follow: AP =  − 2.76 mm, ML =  + 1.8 mm, DV =  − 8.6 mm from bregma. After surgery, animals were placed in a recovery cages and monitored until they were fully awake, then they were returned to their home cages.

### Rotational behavior

Immediately after administration of the tested drugs or vehicle rats were individually placed in automated rotameters (Panlab, Barcelona, Spain) (Ungerstedt 1971; Frau et al. 2013). After a 5-min acclimatization, movements of 90° in clockwise and counter-clockwise directions were recorded for 60 to 120 min. Entire 360° rotations in both directions were counted by computer with 10-min intervals.

### Determination of the concentrations of NA, DA, 5-HT and their metabolites in brain tissue homogenates

The reverse-phase high performance liquid chromatography (HPLC) with the coulometric detection was used to assay tissue concentrations of biogenic amines (NA, DA, 5-HT) and their metabolites: 3,4-dihydroxyphenylacetic acid (DOPAC), homovanillic acid (HVA), 3-methoxytyramine (3-MT) and 5-hydroxyindoleacetic acid (5-HIAA) in the chosen brain structures. Homogenates were prepared from the isolated brain structures (PFC, HIP, STR, SN), separately for the left (lesioned, ipsilateral) and the right (intact, contralateral) side. The procedure was carried out as described previously (Kamińska et al. 2017, 2018). Briefly, tissue samples were weighted, homogenized in ice-cold 0.1 M perchloric acid containing 0.05 mM ascorbic acid and centrifuged (10,000 × g) for 10 min. The supernatants were filtrated using 0.2 μm cellulose filters (Alltech Associates Inc. Deerfeld, IL, USA) and then injected into the HPLC system which consisted of a P680 pump, ASI-100 autosampler and thermostated column compartment TCC-100 (Dionex, Germering, Germany) equipped with C18 Hypersil Gold column (150 × 3.0 mm i.d., 3 μm particle size) fitted with a 10 × 3 mm precolumn (Thermo Fisher Scientific Inc., Waltham, MA, USA). Detection was conducted by means of a Coulochem III detector (ESA Inc., Chelmsford, MA, USA) equipped with a guard cell (ESA 5020) with the electrode set at 600 mV and a dual electrochemical analytic cell (ESA 5010). The applied potential was E1 =  + 350 mV and E2 =  − 220 mV. Temperatures of the analytical cell as well as of the column were maintained at 30 °C. The mobile phase consisted of 35 mM citrate/47 mM disodium phosphate buffer (pH 4.2), supplemented with 0.25 mM EDTA, 0.25 mM 1-octanesulfonic acid sodium salt, 2.4% methanol and 1.3% acetonitrile. The biogenic amines and their metabolites were quantified by comparing the peak area of the tested samples with freshly prepared standards. The obtained data were analyzed using a Chromeleon 6.8 software (Dionex, Germany).

### Statistical analysis

The statistical analysis of the obtained behavioral data (L-DOPA-induced rotations) was performed using the repeated measures analysis of variance (ANOVA) followed by the Newman-Keuls test for post hoc comparisons, when appropriate. The significance of differences in the total number of contralateral rotations (calculated for the entire 2-h session) after administration of the first and last chronic doses of L-DOPA alone or DES + L-DOPA combination was evaluated using Student’s *t* test. The neurochemical data were analyzed using a two-way ANOVA followed by the Newman-Keuls post hoc test, when appropriate. The total rate of DA catabolism was calculated from the ratio of the common DA metabolite HVA to DA concentration and was expressed as an index of the catabolism rate HVA/DA × 100. To assess participation of the MAO-dependent oxidative pathway of DA catabolism, the ratio of DOPAC to DA was calculated and presented as an index (DOPAC/DA) × 100. The catechol-O-methyltransferase (COMT)-dependent methylation pathway was assessed likewise, and the index (3-MT/DA) × 100 was calculated. The total rate of 5-HT catabolism was calculated from the ratio of its metabolite 5-HIAA to 5-HT concentration and was expressed as the catabolism rate index (5-HIAA/5-HT) × 100. The indices were calculated using concentrations from individual tissue samples. The significance of differences between the right and left side of the examined brain structures within each experimental group was estimated by Student’s *t* test for dependent samples.

The *p* values of less than or equal to 0.05 were considered to indicate statistical significance. The statistical analysis was done using STATISTICA 10.0 Software (Statsoft, Inc, USA).

## Results

### The effects of acute and chronic administration of DES and/or L-DOPA on rotational behavior

**Fig. 2:**
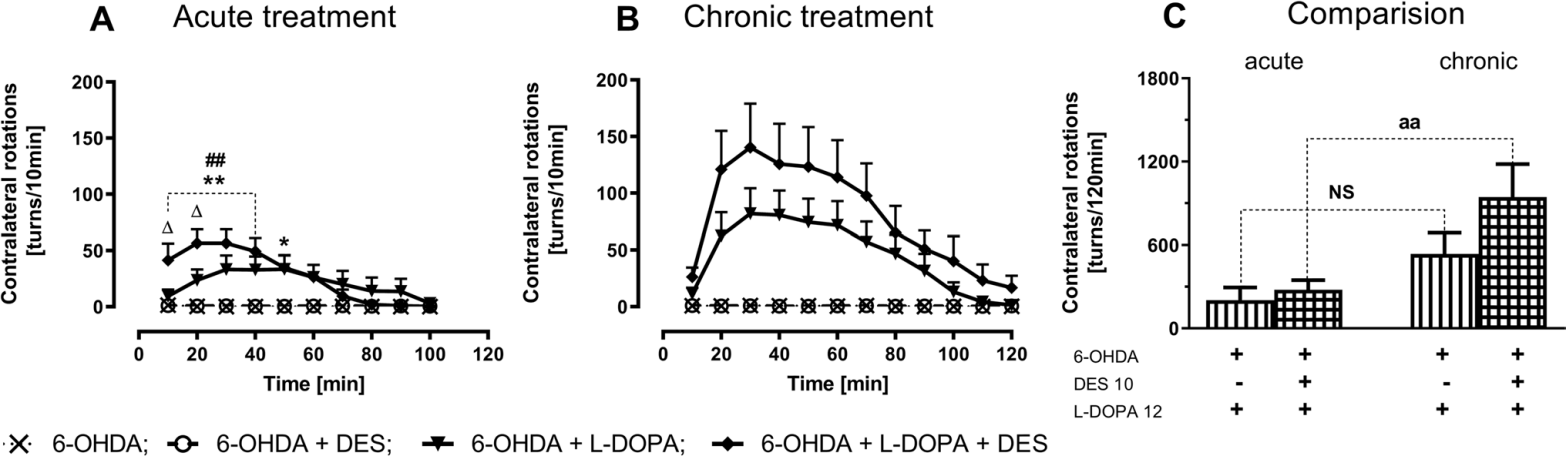
The effects of acute (**A**, **C**) and chronic (**B**, **C**) administration of DES (10 mg/kg) and L-DOPA (12 mg/kg i.p.), alone and in combination, on the level of contralateral rotations in unilaterally 6-OHDA-lesioned rats, measured immediately after the last doses of vehicle, L-DOPA or the DES + L-DOPA combination. Data are presented as the mean ± SEM, numbers of animals in experimental groups *n* = 9–12. Symbols indicate significance of differences according to the post hoc test, ^**^P < 0.01, ^*^P < 0.05 vs. 6-OHDA + veh − ; ^##^*P* < 0.01 vs. 6-OHDA + DES − ; ^∆^*P* < 0.05 vs. 6-OHDA + L-DOPA-treated groups. Letter indicate significance of differences according to Student’s *t* test, ^aa^*P* < 0.01 vs. acute DES + L-DOPA treatment

The time-dependent changes in the number of contralateral rotations in unilaterally 6-OHDA-lesioned rats treated acutely or chronically with vehicle, DES (10 mg/kg) and L-DOPA (12 mg/kg), alone or in combination are presented in Fig. 2A, B. A repeated measures ANOVA performed for acute treatment with DES and L-DOPA revealed a significant treatment effect of L-DOPA *F*(1,37) = 20.174, *P* < 0.0001 and time *F*(9,333) = 16.390, *P* < 0.00001 as well as interactions of time × L-DOPA treatment *F*(9,333) = 16,3001, *P* < 0.0001, time × DES treatment *F*(9,333) = 4.800, *P* < 0.0001, and time × DES × L-DOPA treatment *F*(9,333) = 4.659, *P* < 0.0001 (Fig. 2A).

Acute administration of DES or vehicle did not evoke contralateral rotations in unilaterally 6-OHDA-lesioned rats, in contrast to L-DOPA, which when administered at a single dose of 12 mg/kg, induced pronounced contralateral rotations (Fig. 2A). The number of these rotations gradually increased during the 100-min session, reaching a mean value about 25 turns/10 min between 20 and 60 min of measurement, and then gradually decreased (Fig. 2A). The acute co-administration of DES and L-DOPA, produced a two-phase effect (Fig. 2A). During the first 20 min, the combination of these drugs caused a significant increase in the number of contralateral rotations compared to the effect of a single dose of L-DOPA. Then, a gradual decline was observed, until finally the number of these rotations during the 70–90 min time interval was slightly lower than after a single dose of L-DOPA alone (Fig. 2A). However, as shown in Fig. 2C, there were no significant differences in the total number of contralateral rotations calculated for the entire measurement session in the group of rats receiving L-DOPA alone when compared to the group receiving once the DES + L-DOPA combination.

A repeated measures ANOVA performed for chronic treatment with DES and L-DOPA showed only a significant treatment effect of L-DOPA *F*(1,36) = 33.844, *P* < 0.0001 and time effect *F*(11,396) = 14.871, *P* < 0.0001, as well as an interaction of time × L-DOPA treatment *F*(11,396) = 14.709, *P* < 0.0001 (Fig. 2B). As presented in Fig. 2B, chronic administration of DES or vehicle, similarly as the acute one, did not induce contralateral rotations in unilaterally 6-OHDA-lesioned rats. However, long-term treatment with L-DOPA (12 mg/kg) alone or with the DES + L-DOPA combination resulted in much greater increases in the number of contralateral rotations measured at 10-min intervals (Fig. 2B) than in rats acutely receiving L-DOPA alone or the DES + L-DOPA combination (Fig. 2A). Moreover, chronic combined administration of DES and L-DOPA resulted in a marked upward trend in the number of contralateral rotation compared to the chronic effect of L-DOPA alone (Fig. 2B). The mean value of these rotations between 20 and 60 min of measurement, expressed as a number of turns/10 min, after chronic L-DOPA amounted to about 75 turns/10 min, while after chronic the DES + L-DOPA combination about 125 turns/10 min (Fig. 2B). Furthermore, as shown in Fig. 2C, a comparison of the total number of contralateral rotations after chronic combined treatment with DES + L-DOPA to the number of these rotations after chronic treatment with L-DOPA alone confirms the upward trend illustrated in Fig. 2B. Likewise, comparing of the total number of contralateral rotations after the first dose of L-DOPA versus the number of these rotations after the last chronic dose of L-DOPA shows only an upward trend. On the other hand, the comparison of the total number of contralateral rotations after the first and the last chronic dose of DES + L-DOPA combination shows a significant difference between these groups at the level of *p* < 0.01 (Fig. 2C).

### The effects of chronic administration of DES and/or L-DOPA on the levels of NA in the motor and limbic brain structures

It has been previously shown (Kamińska et al. 2017) that 6-OHDA at a single dose of 16 μg/4 μl injected unilaterally directly into the rat MFB caused drastic declines in NA levels measured 2 weeks after surgery in the STR, HIP and PFC (but not in the SN) on the lesioned (ipsilateral) side when compared to the corresponding side of the sham-operated group. In addition, in the unilaterally 6-OHDA-lesioned group, NA concentrations in all examined brain structures on the intact (contralateral) side remained at nearly the same levels as on the ipsilateral (control) side of the sham-operated group. Therefore, in the present study the contralateral side of 6-OHDA group receiving chronically saline is considered as the control instead of the sham-operated group.

**Fig. 3:**
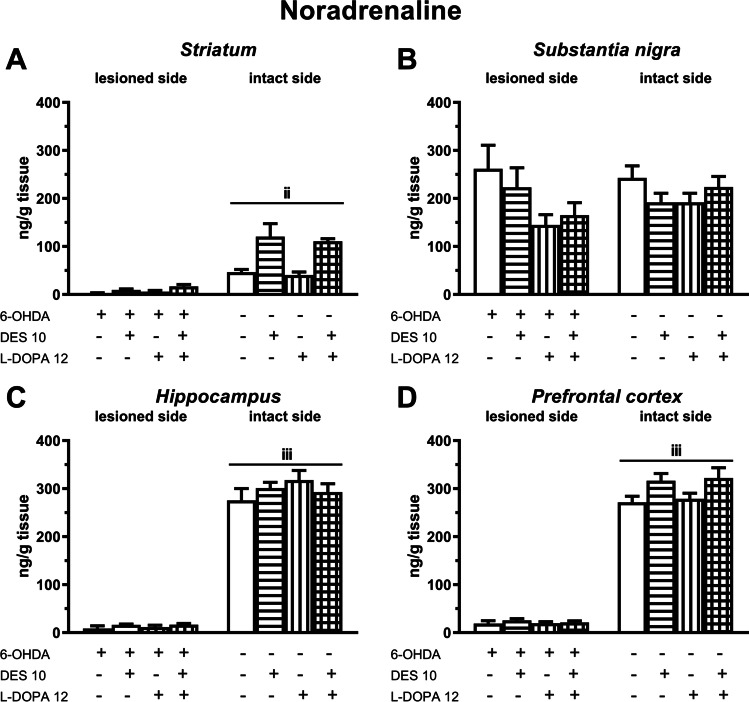
The effects of chronic (21 days) administration of DES (10 mg/kg) and L-DOPA (12 mg/kg i.p.), alone and in combination, on the level of NA in the ipsi- and contralateral striatum (**A**), substantia nigra (**B**), hippocampus (**C**) and prefrontal cortex (**D**) determined 1 h after the last doses of these drugs. Data are presented as the mean ± SEM, numbers of animals in experimental groups *n* = 8–12. Letters indicate significance of differences according to Student’s *t* test, ^iii^*P* < 0.001, ^ii^*P* < 0.01vs. corresponding group on the lesioned side

As to the impact of DES and/or L-DOPA on the NA level in the studied motor brain structures, a two-way ANOVA performed for the ipsilateral STR showed significant treatment effects of L-DOPA (*F*(1,35) = 5.053, *P* < 0.05) and DES (*F*(1,35) = 15.149, *P* < 0.01) but no interaction between these drugs. In the contralateral STR, this analysis revealed only a significant treatment effect of DES (*F*(1,34) = 19.12, *P* < 0.001) but no treatment effect of L-DOPA and interaction between these drugs (Fig. 3A). The above analyses clearly show that in the ipsilateral STR both L-DOPA and DES contribute in the observed increase in NA content, while in the contralateral STR only DES is responsible for this increase (Fig. 3A). In the SN, a two-way ANOVA performed for the NA level in the ipsilateral SN revealed a significant treatment effect of L-DOPA (*F*(1,37) = 5.439, *P* < 0.05), while in the contralateral SN this analysis was non-significant. In the ipsilateral SN, the effect L-DOPA treatment led to a decrease in the NA content in both groups studied (Fig. 3B).

In the limbic brain structures, i.e. in the ipsi- and contralateral HIP and in the ipsilateral PFC, a two-way ANOVA was non-significant. Only in the contralateral PFC, a two-way ANOVA showed a significant treatment effect of DES (*F*(1,35) = 8.307, *P* < 0.01), resulting in an increase in the tissue NA concentration in the groups receiving this drug chronically (Fig. 3D).

### The effects of chronic administration of DES and L-DOPA alone and in combination, on the levels of DA and its metabolites in the motor and limbic brain structures

**Fig. 4:**
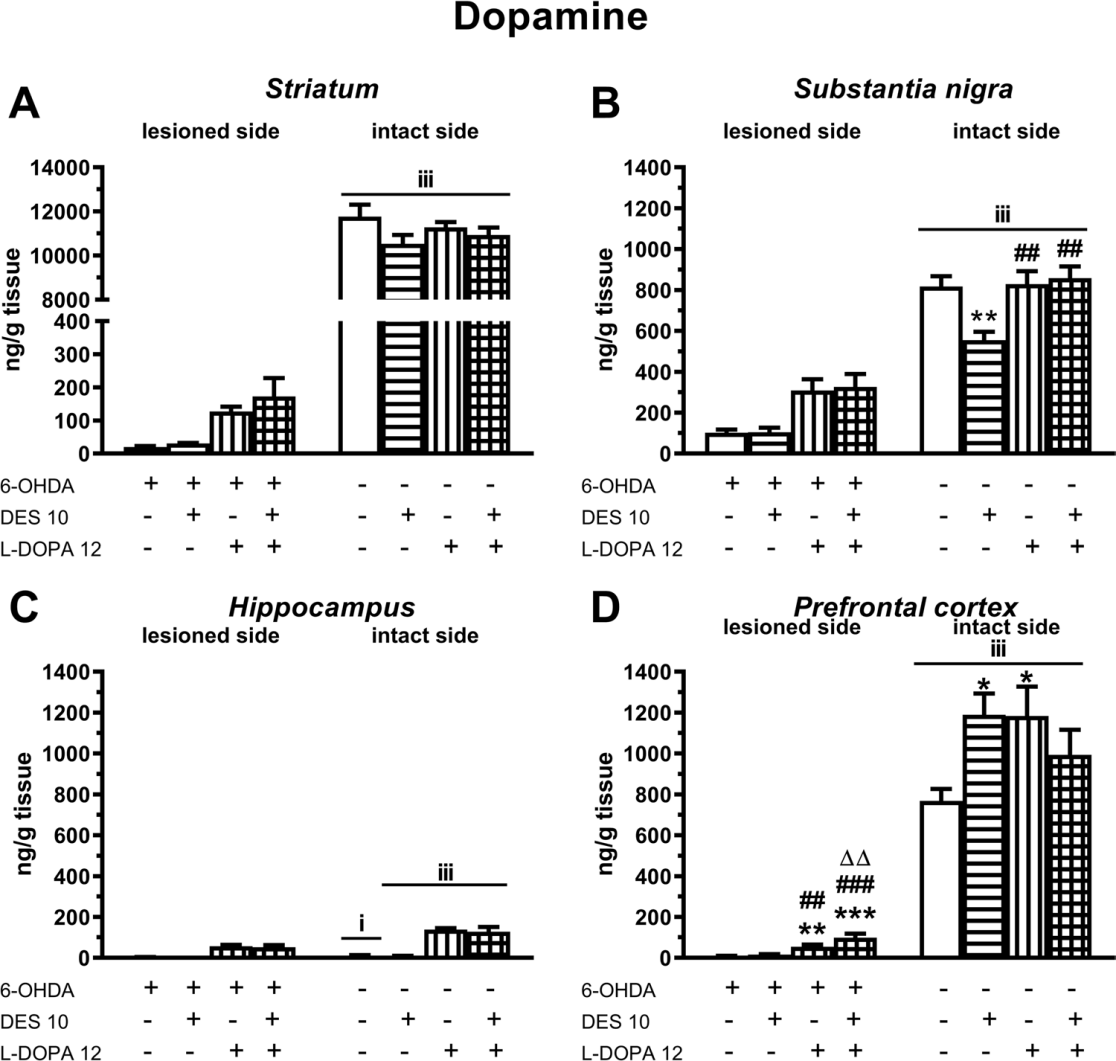
The effects of chronic (21 days) administration of DES (10 mg/kg) and L-DOPA (12 mg/kg i.p.), alone and in combination, on the level of DA in the ipsi- and contralateral striatum (**A**), substantia nigra (**B**), hippocampus (**C**) and prefrontal cortex (**D**) determined 1 h after the last doses of these drugs. Data are presented as the mean ± SEM, numbers of animals in experimental groups *n* = 8–12. Symbols indicate statistically significant differences according to the post hoc test,^***^P < 0.001, ^**^P < 0.01, vs. the 6-OHDA + veh − , ^###^*P* < 0.001, ^##^*P* < 0.01 vs. 6-OHDA + DES − , ^∆∆^*P* < 0.01 vs. 6-OHDA + L-DOPA-treated group on the lesioned or intact side. Letters indicate significance of differences according to Student’s *t* test, ^iii^*P* < 0.001, ^ii^*P* < 0.01, ^i^*P* < 0.05 vs. corresponding group on the lesioned side

A two-way ANOVA performed for DA concentrations (Fig. 4A–C) in the ipsilateral STR, SN and HIP revealed only a significant treatment effect of L-DOPA (for 4A, *F*(1,35) = 27.09, *P* < 0.001; for 4B, *F*(1,37) = 26.60, *P* < 0.001; for 4C, *F*(1,35) = 97.50, *P* < 0.001), a lack of treatment effect of DES and no interaction of DES x L-DOPA. According to the above analysis, as shown in Fig. 1A–C, L-DOPA increased the DA content in the ipsilateral STR, SN and HIP in the tested groups of rats. Only in the ipsilateral PFC (Fig. 4D), a two-way ANOVA demonstrated significant treatment effects of both L-DOPA *F*(1,35) = 46.40, *P* < 0.001 and DES (*F*(1,35) = 7.44, *P* < 0.01), as well as an interaction between these two drugs of a borderline statistical significance (*F*(1,35) = 4.00, *P* = 0.053). A post hoc comparison of the studied groups showed that in the ipsilateral PFC of the rats receiving the DES + L-DOPA combination, the DA content was significantly higher than in the groups receiving the vehicle, DES or L-DOPA alone (Fig. 4D).

Regarding the effect of DES and/or L-DOPA on DA levels in the examined brain motor structures on the contralateral side, a two-way ANOVA performed for the contralateral STR was non-significant (Fig. 4A), while this analysis performed for the contralateral SN, showed significant treatment effects of both L-DOPA (*F*(1,37) = 8.61, *P* < 0.01) and DES (*F*(1,37) = 4.69, *P* < 0.05) and the interaction between these two drugs (*F*(1,37) = 7.33, *P* < 0.05) (Fig. 4B). Post hoc comparison of the tested groups showed that DES alone significantly reduced the endogenous DA content in the contralateral SN, but the combined administration of DES and L-DOPA reversed this effect (Fig. 4B). Finally, a two-way ANOVA performed for DA content in the contralateral PFC demonstrated a significant interaction of DES × L-DOPA (*F*(1,35) = 7.70, *P* < 0.01) (Fig. 4D). Post hoc comparison of the studied groups showed that both DES and L-DOPA significantly increased DA content in the contralateral PFC, but their combined administration was less effective (Fig. 4D).

As to DA catabolism in the motor and limbic brain structures, a two-way ANOVA revealed a significant treatment effect of L-DOPA on the levels of DA metabolites, i.e. the intraneuronal DA metabolite DOPAC and the total DA metabolite HVA in the ipsilateral STR as well as in the ipsi- and contralateral SN, HIP and PFC (Tables 1 and 2). In the abovementioned brain structures, L-DOPA increased the levels of both these DA metabolites. Unlike L-DOPA treatment, a two-way ANOVA showed no effect of DES treatment on DOPAC and HVA levels in the examined brain structures, except for the contralateral STR and the ipsilateral HIP (Tables 1 and 2). In the contralateral STR, a two-way ANOVA performed for the DOPAC level showed a significant treatment effect of DES and an interaction between DES and L-DOPA (Table 1). In the ipsilateral HIP, in addition to the therapeutic effects of L-DOPA and DES on the HVA level, there was also a significant interaction between these drugs, and a post hoc comparison showed that under the conditions of combined administration of DES + L-DOPA, the HVA content was significantly lower than after L-DOPA alone (Table 2). Regarding the impact of L-DOPA and DES on the level of the extracellular DA metabolite 3-MT, the two-way ANOVA showed significant therapeutic effects of both these drugs in the ipsi- and contralateral STR, indicating that their administration resulted in increases in the concentration of 3-MT. Compared to the STR only in the ipsilateral SN, this analysis revealed a significant treatment effect of L-DOPA and an interaction of DES and L-DOPA (Table 1). In the latter brain structure, after chronic administration of L-DOPA alone or the DES + L-DOPA combination, significant increases in the content of 3-MT were observed (Table 1).

**Table 1:** The effect of 21-day treatment with desipramine (DES; 10 mg/kg) and/or L-DOPA (12 mg/kg), alone and in combination, on the levels of dopamine metabolites (DOPAC, 3-MT, HVA) in the striatum (STR) and substantia nigra (SN) of unilaterally 6-OHDA-lesioned rats

Brain structures	DOPAC (ng/g tissue)		3-MT (ng/g tissue)		HVA(ng/g tissue)	
	Ipsilateral side 6-OHDA(L)	Contralateral side Intact	Ipsilateral side 6-OHDA(L)	Contralateral side Intact	Ipsilateral side 6-OHDA(L)	Contralateral side Intact
***Striatum***						
L + veh	11.7 ± 1.3	1742 ± 190^iii^	3.2 ± 0.5	335 ± 19^iii^	3.8 ± 0.3	714 ± 40^iii^
L + DES	7.4 ± 1	967 ± 42^**,iii^	8.2 ± 1	274 ± 19^iii^	5.6 ± 0.7	522 ± 55^iii^
L + L-DOPA	179 ± 17	1579 ± 152^##,iii^	11.1 ± 1.7	300 ± 19^iii^	176 ± 17	1058 ± 93^iii^
L + DES + L-DOPA	160 ± 38	1414 ± 142^#,iii^	13.8 ± 2.4	221 ± 12^iii^	137 ± 37	960 ± 103^iii^
*Effect of L-DOPA*	*F(1,35)* = *85.1, P* < *0.001*	*No*	*F(1,35)* = *22.3, P* < *0.001*	*F(1,34)* = *5.9, P* < *0.05*	*F(1,35)* = *79.2, P* < *0.001*	*F(1,34)* = *28.0, P* < *0.001*
*Effect of DES*	*No*	*F(1,34)* = *11.3, P* < *0.01*	*F(1,35)* = *7.3, P* < *0.05*	*F(1,34)* = *14.9, P* < *0.001*	*No*	*No*
*Interaction*	*No*	*F(1,34)* = *4.75, P* < *0.05*	*No*	*No*	*No*	*No*
***Substantia nigra***						
L + veh	17.1 ± 2.4	176 ± 20^iii^	3.4 ± 0.8	27.5 ± 8^i^	4.7 ± 1.1	72 ± 8^iii^
L + DES	1 ± 0.4	75 ± 7^iii^	15.7 ± 1.7	24.5 ± 2^i^	9.0 ± 0.8	51 ± 4^iii^
L + L-DOPA	172 ± 36	352 ± 53^iii^	27.7 ± 4^***,#^	31.0 ± 2.9	161 ± 31	269 ± 45^iii^
L + DES + L-DOPA	182 ± 47	393 ± 77^iii^	26.7 ± 3^***^	29.9 ± 3.1	179 ± 49	318 ± 73^ii^
*Effect of L-DOPA*	*F(1,37)* = *39.0, P* < *0.001*	*F(1,37)* = *33.2, P* < *0.001*	*F(1,37)* = *37.1, P* < *0.001*	*No*	*F(1,37)* = *40.2, P* < *0.001*	*F(1,37)* = *37.5, P* < *0.001*
*Effect of DES*	*No*	*No*	*No*	*No*	*No*	*No*
*Interaction*	*No*	*No*	*F(1,37)* = *11.5, P* < *0.01*	*No*	*No*	*No*

One hour after administration of the last doses of the tested drugs, the rats were sacrificed, and the ipsi- and contralateral STR and SN tissue samples were separately dissected from their brains. The data are presented as the mean ± S.E.M.; the number of rats per group was *n* = 8–12. Significance of differences in paired Student’s *t* test ^i^*P* < 0.05, ^ii^*P* < 0.01, ^iii^*P* < 0.001 vs. ipsilateral side of respective group. Statistical significance of differences between all examined groups in the STR and SN was calculated using a two-way ANOVA followed by the Newman-Keuls test when appropriate, ^**^P < 0.01, ^***^P < 0.001 vs. L + veh-treated group, ^#^*P* < 0.05, ^##^*P* < 0.01 vs. L + DES-treated group of corresponding ipsi- or contralateral sides

**Table 2:** The effect of 21-day treatment with desipramine (DES; 10 mg/kg) and/or L-DOPA (12 mg/kg), alone and in combination, on the levels of dopamine metabolites in the prefrontal cortex (PFC) and hippocampus (HIP) of unilaterally 6-OHDA-lesioned rats

Brain structures	DOPAC (ng/g tissue)		3-MT (ng/g tissue)		HVA (ng/g tissue)	
	Ipsilateral side Veh/6-OHDA(L)	Contralateral side Intact	Ipsilateral side Veh/6-OHDA(L)	Contralateral side Intact	Ipsilateral side 6-OHDA(L)	Contralateral side Intact
***Prefrontal cortex***						
L + veh	3.4 ± 0.7	151 ± 17^iii^	1.7 ± 0.2	20.2 ± 2.2^iii^	3.7 ± 0.5	81 ± 6^iii^
L + DES	3.0 ± 0.5	161 ± 12^iii^	5.3 ± 0.7	33.0 ± 2.2^iii^	4.7 ± 0.4	91 ± 12^iii^
L + L-DOPA	82 ± 17	297 ± 41^iii^	12.0 ± 3.3^***,#^	47.9 ± 8.3^***,iii^	113 ± 21	271 ± 40^iii^
L + DES + L-DOPA	123 ± 27	306 ± 47^iii^	6.2 ± 0.7	22.7 ± 2.1^∆,iii^	137 ± 31	286 ± 52^iii^
*Effect of L-DOPA*	*F(1,35)* = *54.8, P* < *0.001*	*F(1,35)* = *24.2, P* < *0.001*	*F(1,35)* = *11.7, P* < *0.001*	*No*	*F(1,35)* = *57.0, P* < *0.001*	*F(1,35)* = *42.9, P* < *0.001*
*Effect of DES*	*No*	*No*	*No*	*No*	*No*	*No*
*Interaction*	*No*	*No*	*F(1,35)* = *7.9, P* < *0.01*	*F(1,35)* = *19.0, P* < *0.001*	*No*	*No*
***Hippocampus***						
L + veh	5.5 ± 0.8	7.1 ± 0.7	2.4 ± 0.4	3.7 ± 0.8	2.7 ± 0.5	3.5 ± 0.4
L + DES	0.4 ± 0.1	1.8 ± 0.1^iii^	3.7 ± 0.8	3.2 ± 0.6	1.2 ± 0.2	1.7 ± 0.5
L + L-DOPA	94 ± 9	135 ± 9^ii^	6.1 ± 0.9	7.3 ± 1.3	142 ± 12^***,###^	156 ± 10
L + DES + L-DOPA	83 ± 20	115 ± 27^i^	5.4 ± 1.0	6.8 ± 1.5	89 ± 21^***,###,∆∆∆^	117 ± 29^i^
*Effect of L-DOPA*	*F(1,35)* = *88.5, P* < *0.001*	*F(1,35)* = *102.4, P* < *0.001*	*F(1,35)* = *11.2, P* < *0.01*	*F(1,35)* = *12.8, P* < *0.01*	*F(1,35)* = *122.7, P* < *0.001*	*F(1,35)* = *111.7, P* < *0.001*
*Effect of DES*	*No*	*No*	*No*	*No*	*F(1,35)* = *7.1, P* < *0.05*	*No*
*Interaction*	*No*	*No*	*No*	*No*	*F(1,35)* = *6.3, P* < *0.05*	*No*

One hour after administration of the last doses of the tested drugs, the rats were sacrificed, and the ipsi- and contralateral PFC and HIP tissue samples were separately dissected from their brains. The data are presented as the mean ± S.E.M.; the number of rats per group was *n* = 8–12. Significance of differences in paired Student’s *t* test ^i^*P* < 0.05, ^ii^*P* < 0.01, ^iii^*P* < 0.001 vs. ipsilateral side of respective group. Statistical significance of differences between all examined groups in the PFC and HIP was calculated using a two-way ANOVA followed by the Newman-Keuls test when appropriate, ^***^P < 0.001 vs. L + veh-treated group, ^#^*P* < 0.05, ^###^*P* < 0.001 vs. L + DES-treated group, ^∆^*P* < 0.05, ^∆∆∆^*P* < 0.001 vs. L + L-DOPA-treated group of corresponding ipsi- or contralateral sides

Regarding the level of 3-MT in the examined limbic brain structures, the two-way ANOVA showed a significant treatment effect of L-DOPA in the ipsilateral PFC and in the ipsi-and contralateral HIP, but no treatment effect of DES in both these structures (Table 2). In these structures, L-DOPA increased the level of 3-MT. However, only in the PFC, there was a significant interaction between DES and L-DOPA. Post hoc comparison of the examined groups in the ipsi- or contralateral PFC demonstrated that the combined administration of DES and L-DOPA reduced the level of 3-MT compared to its content in the group treated with L-DOPA alone (Table 2).

Detailed analysis of DA turnover as measured by the metabolic ratios of DOPAC/DA, 3-MT/DA and HVA/DA is presented in Tables 4 and 5 in the supplementary materials.

### The effects of chronic administration of DES and L-DOPA alone and in combination, on the level of 5-HT and its metabolite in the motor and limbic brain structures

**Fig. 5:**
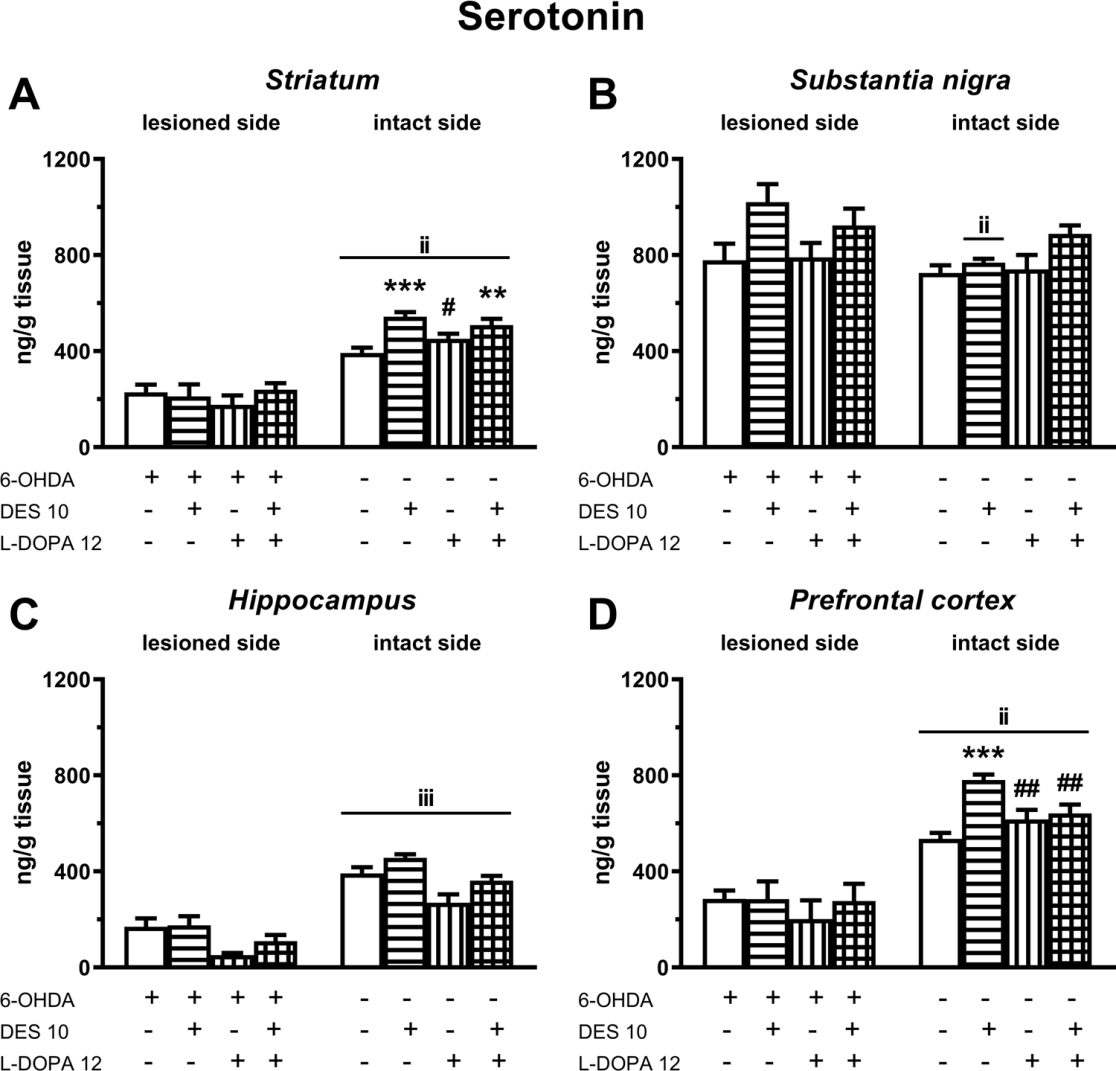
The effects of chronic (21 days) administration of DES (10 mg/kg) and L-DOPA (12 mg/kg i.p.), alone and in combination, on the level of 5-HT in the ipsi- and contralateral striatum (**A**), substantia nigra (**B**), hippocampus (**C**) and prefrontal cortex (**D**) determined 1 h after the last doses of these drugs. Data are presented as the mean ± SEM, numbers of animals in experimental groups *n* = 8–12. Symbols indicate statistically significant differences according to the Newman-Keuls post hoc test, ^*^P < 0.05, ^**^P < 0.01, ^***^P < 0.001 vs. the 6-OHDA + veh − , ^#^*P* < 0.05, ^##^*P* < 0.01, ^###^*P* < 0.001 vs. 6-OHDA + DES − , ^∆^*P* < 0.05 vs. 6-OHDA + L-DOPA-treated group of lesioned or intact side

As to the effects of DES and L-DOPA on 5-HT levels in the motor structures of the rat brain, a two-way ANOVA performed for the ipsilateral STR was non-significant, while in the ipsilateral SN this analysis showed only a significant treatment effect of DES (*F*(1,37) = 7.043, *P* < 0.05). In the latter structure, DES increased 5-HT level in the studied groups (Fig. 5B). With regard to the influence of the tested drugs in the limbic structures of the brain, a two-way ANOVA performed for the ipsilateral HIP revealed a significant treatment effect of L-DOPA (*F*(1,35) = 7.987, *P* < 0.01), while in the ipsilateral PFC this analysis was non-significant. In the ipsilateral HIP, L-DOPA caused a decrease in the level of 5-HT in the studied groups (Fig. 5C).

However, on the contralateral side both treatment effects and interactions between these drugs were distinctly visible in all studied structures. Consistently, in the contralateral STR a two-way ANOVA performed for 5-HT content demonstrated a significant treatment effect of DES (*F*(1,34) = 21.690, *P* < 0.001) and an interaction of DES × L-DOPA (*F*(1,34) = 4.436, *P* < 0.05). In this structure, post hoc comparison showed that DES increased 5-HT level in the studied group, although this effect was slightly weaker when DES was given in combination with L-DOPA (Fig. 5A). In the contralateral SN a two-way ANOVA revealed only a significant treatment effect of DES (*F*(1,37) = 5.701, *P* < 0.05). DES caused an increase in the 5-HT content in this structure (Fig. 4B).

As to the 5-HT level in the limbic structures, in the contralateral HIP a two-way ANOVA showed treatment effects of L-DOPA (*F*(1,35) = 19.049, *P* < 0.001) and DES (*F*(1,35) = 9.794, *P* < 0.01) but there was no interaction between these drugs (Fig. 5C). In this structure L-DOPA decreased 5-HT level while DES increased it (Fig. 5C). In the contralateral PFC, a two-way ANOVA demonstrated a significant treatment effect of DES (*F*(1,35) = 18.987, *P* < 0.001) and an interaction of DES × L-DOPA (*F*(1,35) = 12.763, *P* < 0.01) (Fig. 5D). As shown by the post hoc comparison of the tested groups, in the latter brain structure DES alone increased the level of 5-HT, while L-DOPA administered both alone and in combination with DES lowered it (Fig. 5D).

As to 5-HT catabolism in the motor and limbic brain structures, a two-way ANOVA revealed a significant treatment effect of L-DOPA on the levels of 5-HIAA on the ipsilateral side only in the SN and HIP while on the contralateral side in the STR, SN and PFC (Table 3). This analysis also showed a treatment effect of DES only in the contralateral PFC and HIP. Regarding 5-HT turnover assessed as the 5-HIAA/5-HT metabolic ratio, a two-way ANOVA showed significant treatment effects for both L-DOPA and DES in the ipsilateral STR, PFC and HIP, while in the ipsilateral SN this analysis only revealed therapeutic effect of L-DOPA. Interestingly, in the ipsilateral PFC and HIP significant interactions were also observed between DES and L-DOPA. In the latter structures, post hoc comparison of the examined groups showed that combined administration of DES + L-DOPA decreased turnover of 5-HT measured as 5-HIAA/5-HT metabolic index when compared to the value of this index in the group treated with L-DOPA alone (Table 3).

**Table 3:** The effect of 21-day treatment with desipramine (DES; 10 mg/kg) and/or L-DOPA (12 mg/kg), alone and in combination for 3 weeks, on the level of 5-HT metabolite (5-HIAA) and on 5-HT turnover assessed as metabolic index of 5-HIAA/5-HT in the motor (striatum, substantia nigra) and limbic (prefrontal cortex, hippocampus) brain structures of unilaterally 6-OHDA-lesioned rats

Brain structures	5-HIAA (ng/g tissue)		5-HIAA/5-HT	
	Ipsilateral side 6-OHDA(L)	Contralateral side Intact	Ipsilateral side 6-OHDA(L)	Contralateral side Intact
***Striatum***				
L + veh	291 ± 48	426 ± 19^i^	128 ± 10	110 ± 5
L + DES	209 ± 40	411 ± 20^iii^	115 ± 8	76 ± 3^***,iii^
L + L-DOPA	288 ± 64	448 ± 14	175 ± 19	102 ± 6^###,ii^
L + DES + L-DOPA	278 ± 49	473 ± 26^ii^	135 ± 13	93 ± 4^*,##,i^
*Effect of L-DOPA*	*No*	*F(1,34)* = *4.4, P* < *0.05*	*F(1,35)* = *6.9, P* < *0.05*	*No*
*Effect of DES*	*No*	No	*F(1,35)* = *4.2, P* < *0.05*	*F(1,34)* = *22.6, P* < *0.001*
*Interaction*	*No*	*No*	No	*F(1,34)* = *8,6, P* < *0.01*
***Substantia nigra***				
L + veh	409 ± 13	433 ± 15	57 ± 4	61 ± 3
L + DES	410 ± 23	378 ± 9	41 ± 2	49 ± 1^*,ii^
L + L-DOPA	480 ± 39	431 ± 29	66 ± 9	60 ± 3^#^
L + DES + L-DOPA	559 ± 26	561 ± 57^**,###,∆∆^	62 ± 4	63 ± 6^#^
*Effect of L-DOPA*	*F(1,37)* = *15.5,* < *0.001*	*F(1,37)* = *10.1, P* < *0.01*	*F(1,37)* = *6.5,P* < *0.05*	*F(1,37)* = *4.2, P* < *0.05*
*Effect of DES*	*No*	*No*	*No*	*No*
*Interaction*	*No*	*F(1,37)* = *10.4, P* < *0.01*	*No*	*F(1,37)* = *5.3, P* < *0.05*
***Prefrontal cortex***				
L + veh	142 ± 17	230 ± 9^ii^	50 ± 3	44 ± 2^ii^
L + DES	103 ± 22	204 ± 10^iii^	43 ± 4	26 ± 1^ii^
L + L-DOPA	108 ± 17	282 ± 18^iii^	131 ± 35^**,##^	48 ± 5^i^
L + DES + L-DOPA	118 ± 25	226 ± 8^ii^	49 ± 4^∆∆^	36 ± 2^ii^
*Effect of L-DOPA*	*No*	*F(1,35)* = *9.2,* < *0.01*	*F(1,35)* = *6.7, P* < *0.05*	*F(1,35)* = *6.4, P* < *0.05*
*Effect of DES*	*No*	*F(1,35)* = *11.8,* < *0.01*	*F(1,35)* = *7.3, P* < *0.05*	*F(1,35)* = *27.9, P* < *0.001*
*Interaction*	*No*	*No*	*F(1,35)* = *5.2, P* < *0.05*	*No*
***Hippocampus***				
L + veh	218 ± 30	367 ± 17^ii^	160 ± 19	96 ± 4^ii^
L + DES	155 ± 22	272 ± 9^***,iii^	107 ± 9	60 ± 4^iii^
L + L-DOPA	134 ± 10	314 ± 10^**,iii^	316 ± 46^***,###^	140 ± 23^ii^
L + DES + L-DOPA	133 ± 16	286 ± 18^***,iii^	151 ± 18^∆∆∆^	80 ± 4^ii^
*Effect of L-DOPA*	*F(1,35)* = *5.7, P* < *0.05*	*No*	*F(1,35)* = *15,5, P* < *0.001*	*F(1,35)* = *8.2, P* < *0.01*
*Effect of DES*	*No*	*F(1,35)* = *23.3,P* < *0.001*	*F(1,35)* = *18.7, P* < *0.001*	*F(1,35)* = *18.2, P* < *0.001*
*Interaction*	*No*	*F(1,35)* = *6.9, P* < *0.05*	*F(1,35)* = *4.9, P* < *0.05*	*No*

One hour after administration of the last doses of the tested drugs, the rats were sacrificed, and the ipsi- and contralateral STR, SN, PFC and HIP tissue samples were separately dissected from their brains. The data are presented as the mean ± S.E.M.; the number of rats per group was *n* = 8–12. Significance of differences in paired Student’s *t* test ^i^*P* < 0.05, ^ii^*P* < 0.01, ^iii^*P* < 0.001 vs. ipsilateral side of the respective group. Statistical significance of differences between all examined groups in the STR, SN, PFC and HIP was calculated using a two-way ANOVA followed by the Newman-Keuls test when appropriate, ^*^P < 0.05, ^**^P < 0.01, ^***^P < 0.001 vs. L + veh-treated group, ^#^*P* < 0.05, ^##^*P* < 0.01, ^###^*P* < 0.001vs. L + DES-treated group, ^∆∆^*P* < 0.01, ^∆∆∆^*P* < 0.001 vs. L + L-DOPA-treated group of corresponding ipsi- or contralateral sides

## Discussion

In this study, we assessed the effects of long-term treatment with the tricyclic antidepressant DES and the widely used anti-Parkinsonian drug L-DOPA, either alone or in combination, based on monoamine levels (NA, DA, 5-HT) and their metabolism in motor (STR, SN) and limbic (PFC, HIP) brain structures, in rats with unilateral damage to the monoaminergic pathways caused by the injection of 6-OHDA into the MFB (Kamińska et al. 2017). The consequences of these neurochemical changes were analyzed in the context of locomotor activity measured as rotational behavior and the potential mental disorders occurring in PD.

### Impact of DES and L-DOPA on motor functions

In the present study, unilateral injection of 6-OHDA at a dose of 16 μg/4 μl into the rat MFB caused extensive degeneration of the nigrostriatal DA neurons, eventually leading to a functional imbalance between the two striata (Kamińska et al.^2017^, ^2018^). Chronic administration of the DA precursor L-DOPA is the most effective symptomatic treatment of motor deficits in the majority of PD patients and in the animal models of this disease. The motor feature most commonly described in unilaterally 6-OHDA-lesioned rats treated chronically with L-DOPA, namely pronounced turning behavior towards the side contralateral to the lesion, occurs already after the first dose of this drug and gradually increases with the prolongation of treatment. The intensity of these rotations is directly dependent on the supersensitivity of the striatal postsynaptic dopamine (DA) receptors, which is a result of DA depletion on the lesioned side, and depends on the dose of L-DOPA used (Schwarting and Huston 1996, Lindgren et al. 2007, Duty and Jenner 2011). It was widely believed that the ability of L-DOPA to induce contralateral rotations in unilaterally 6-OHDA-lesioned rats was a valuable marker reflecting antiparkinsonian activity of this drug (Schwarting and Huston 1996; Duty and Jenner 2011), although nowadays contralateral rotations are considered rather as a marker of locomotor activity. Thus, the contralateral turning responses constitute a useful test of predictive validity for screening new drugs, providing a means for qualitative and quantitative assessment of their therapeutic efficacy in motor stimulation.

On the other hand, a long-term treatment with L-DOPA leads to development of AIMs which are also a consequence of slowly developing sensitization (Carta et al. 2006; Lindgren et al. 2007). AIMs can be classified into 3 major subtypes, including abnormal movement of the neck-trunk (axial AIMs), forelimbs (limb AIMs) and abnormal jaw movements and tongue protrusion (orolingual AIMs)(Lindgren et al. 2007). In order to induce the stable level of dyskinesias, rats with unilateral 6-OHDA lesion were primed with L-DOPA in the dose range of 3–20 mg/kg usually for 1 to 3 weeks (Lundblad et al. 2002, 2005, Lindgren et al. 2007, Dekundy et al. 2007, Bishop et al. 2012). Some researchers attempted to link the occurrence of L-DOPA-induced dyskinesias in 6-OHDA-lesioned rats with changes in the expression of monoamine transporters (Chatibut et al. 2012, Conti et al. 2016a). According to the study by Chatibut et al. (2012), unilaterally 6-OHDA-lesioned rats in which the loss of dopamine transporter (DAT), determined using western blot technique, was greater than or equal to 70%, showed a significant increase in the expression of NET in the ipsilateral STR. Since NET has the ability of synaptic DA uptake under conditions of a severe DAT loss (Arai et al. 2008), it has been postulated that blocking the striatal NET activity by selective inhibitors, such as DES, and thus increasing the extracellular level of the L-DOPA-derived DA may reveal therapeutic potential and also may cause side effects. In terms of therapeutic potential, an increase in the extracellular DA concentration can lead to locomotor benefits, while overstimulation of postsynaptic DA receptors may worsen AIMs. In support of the latter view, another study by Chotibut et al. (2014) showed that the global level of AIMs in a group of rats that, starting on day 9 after a unilateral injection of 6-OHDA into the MFB, were first administered DES (12 mg/kg) alone for 10 days, followed by the drug combination of DES (12 mg/kg) + L-DOPA (6 mg/kg) for 20 consecutive days, was significantly higher than in the group of rats which were treated analogously first with the vehicle and then with the vehicle + L-DOPA combination. On the other hand, since administration of DES alone in the same regimen as L-DOPA alone did not induce dyskinesias (Chotibut et al. 2014), it became clear that without L-DOPA, DES did not have the ability to induce dyskinesias. These results also suggest that pretreatment of 6-OHDA-lesioned rats with DES alone, before the combined administration of DES + L-DOPA, may lead to much stronger stimulation of postsynaptic DA receptors than after L-DOPA alone. Finally, it is worth adding that in the studies cited above, no changes in the tissue concentration of NA between the ipsi- and contralateral STR were observed, what was interpreted as the lack of damage in the striatal noradrenergic innervation after unilateral 6-OHDA administration into the MFB. The latter effect remains in contrast to the significant degeneration of noradrenergic innervation observed in PD (Braak et al. 2003, 2004; Zarow et al. 2003; Del Tredici and Braak 2012, 2013).

Contrary to the research by Chatibut et al. (2012, Chotibut et al. 2014), rats with unilateral 6-OHDA lesion showed no significant changes in the expression of NET protein in the ipsilateral STR compared to sham-operated controls in the studies by Conti et al. (2016a, b). In these rats, no changes in the content of NA between intact and lesioned striatal tissue were also observed (Conti et al. 2016a). In both these studies, 6-OHDA lesioned rats were primed with L-DOPA (12 or 6 mg/kg) to induce a stable level of AIMs. In the model of dyskinesias established in this way, it was demonstrated that the NET blocker nisoxetine (5, 10 mg/kg) only mildly exacerbated AIMs, but also mildly promoted locomotor behavior assessed in the forepaw adjustment test (FAS) (Conti et al. 2016a, b). In the subsequent study, Conti et al. (2016a) showed that DES at doses of 7.5, 15 and 30 mg/kg did not exacerbate L-DOPA (6 mg/kg)-induced AIMs but deferred them to later time points in testing. However, higher doses of DES (15 and 30 mg/kg) reduced rotational behavior and the number of steps in the FAS test.

In our study, the priming procedure was not used prior to the combined administration of DES + L-DOPA; hence, it is difficult to predict whether this method of administration of the tested drugs may be relevant in the context of reducing AIM expression. However, in our previous (Kamińska et al. 2018) and current study, contrary to that by Conti et al. (2016a), infusion of 6-OHDA directly into the MFB during stereotaxic surgery was not preceded by intraperitoneal administration of DES (25 mg/kg). Thus, both the drastic reduction in NA content in the ipsilateral STR, and the previously described reduction in the specific [^3^H] nisoxetine binding to NET in this structure analyzed by autoradiography, may have been caused by the 6-OHDA-induced severe damage of the ascending noradrenergic axons, analogous to the drastic loss of DA in the ipsilateral STR and reduction of [^3^H] GBR 12,935 binding to DAT in this structure, as a result of severe damage to the nigrostratal dopaminergic pathway by this neurotoxin (Kamińska et al. 2018). Hence, it seems that in our model, blockade of the striatal NET by DES in the presence of L-DOPA could not have had a major impact on the increase in extracellular DA levels, and thus on the intensity of AIMs. In contrast to the ipsilateral STR, there was no decrease in the NA content in the ipsilateral SN, while binding of [^3^H] nisoxetine to NET significantly increased (Kamińska et al. 2018), suggesting that noradrenergic innervation of the ipsilateral SN was not damaged by 6-OHDA injected into the MFB. The increases in the NET binding in the ipsilateral SN might result from an increased affinity of NET for [^3^H] nisoxetine or from an up-regulation in the number of NET binding sites in this structure. Therefore, it is reasonable to suppose that the blockade of NET by DES could increase L-DOPA-derived extracellular DA level in the ipsilateral SN in our study. This assumption is supported by the study of Navailles et al. (2014), who by microdialysis showed that administration of DES at a dose of 10 mg/kg in combination with 12 mg/kg L-DOPA, just as in our study, did not change the extracellular DA level in the ipsilateral STR, but at least doubled its level in the ipsilateral substantia nigra pars reticulata (SNr). The latter data are important in the context of the therapeutic effect of the combined treatment with DES + L-DOPA, as the increased extracellular DA level in the SNr and following activation of the nigral D1 receptors is associated with the production of contralateral rotation (Robertson and Robertson 1989), and thus with the maintenance of motility.

For a long time some authors believed that L-DOPA-induced contralateral rotations in rats and mice could be used as a measure of dyskinesia (Henry et al. 1998; Papa et al. 1994). However, because long-acting dopaminergic drugs, such as bromocriptine, exhibit a very high rotational sensitization and a very low dyskinesiogenic potential, only AIMs provide a specific measure of dyskinesia (Carta et al. 2006, Lindgren et al. 2007). On the other hand, compounds that modulate serotonergic transmission, such as 5-HT_1A/1B_ agonists, have been shown to suppress L-DOPA-induced AIMs in animal models of PD, but at the same time they reduced the stimulating effect of L-DOPA on motility (Pinna et al. 2016). However, more recent studies show that rotational behavior can only be used as an indicator of L-DOPA-induced locomotor activity (Tronci and Francardo 2018), especially because amantadine, the only anti-diskinetic drug used in the clinic (Perez-Lloret and Rascol 2018), and genetic interventions aimed to reduce the severity of dyskinesia (i.e. RasGRF1 inactivation) (Fasano et al. 2010; Lundblad et al. 2005) had no effect on the number of contralateral rotations.

In our study, chronic administration of L-DOPA at a dose of 12 mg/kg resulted in a gradual increase in the number of contralateral rotations compared to the effect of the first dose of this drug, while DES alone, administered both acutely or chronically, had no effect on rotational behavior. However, chronic treatment with the L-DOPA + DES combination induced a stronger upward trend in the number of contralateral rotations than chronic administration of L-DOPA alone, over 2-h measurement period, despite almost the same increases in the tissue concentrations of L-DOPA-derived DA in the ipsilateral STR and SN in both these groups. Interestingly, the first combined dose of L-DOPA + DES caused a significantly greater increase in the number of contralateral rotations only during the first 40 min of measurement; however, in the next 60 min, a much faster decrease in their number was observed than after L-DOPA alone. The data analysis presented above suggests that the mechanisms underlying motor behavior after acute administration of these drugs are different from that after chronic treatment.

As for the noradrenergic innervation of various brain structures, it is well known that the LC is the primary source of an extensive, but regionally specialized, noradrenergic forebrain innervation (Benarroch 2018). This tiny brainstem structure is the only source of NA for the HIP and neocortex, the regions critical for higher cognitive and affective processes (Xing et al. 2016; Takeuchi et al. 2016). When considering the noradrenergic innervation of the basal ganglia nuclei that control motor functions, it is worth emphasizing that the substantia nigra pars compacta (SNc) is one of the most heavily innervated structures unlike the SNr, which receives only sparse NA projections from the LC (Jones and Moore 1977). The STR is almost devoid of NA afferents, but the striatal subfield, i.e. the shell sub-region of the nucleus accumbens (NAcc), receives moderately dense noradrenergic innervation, although most of it arises from the nucleus tractus solitarius (NTS; A2 region) and to a much lesser extent from the caudal ventrolateral medulla (CVLM; A1 region) or the LC (Berrdige et al. 1997, Delfs et al. 1998).

As described previously, in the rat model of PD used in this study (Kamińska et al. 2017), the unilateral injection of 6-OHDA into the MFB without DES pretreatment before surgery severely damaged the noradrenergic pathways innervating the ipsilateral structures of the forebrain, such as STR, PFC, HIP, which resulted in drastic drops in their NA concentrations but such a decrease in the NA content was not found in the SN. Chronic treatment with DES alone did not significantly increase the tissue NA levels in any of the examined ipsilateral brain structures. However, in the ipsilateral STR, a significant treatment effect of both L-DOPA and DES on the NA level was observed. In the latter structure combined administration of DES + L-DOPA increased the NA level. Moreover, in the contralateral STR, a significant treatment effect of DES on the level of NA was found. It seems, that the noradrenergic pathways innervating the striatal subfield, i.e. the NAcc shell, originating mainly from the A1 and A2 regions (Delfs et al. 1998; Manz et al. 2021), which could not be damaged by the unilateral administration of 6-OHDA into the left MFB, played a significant role in increasing the NA content in the ipsilateral STR. Thus, DES when administered in combination with L-DOPA could increase the level of NA, due to inhibition of its reuptake by NET at the terminals of the ascending noradrenergic pathways from regions A1 and A2, but it cannot be ruled out that L-DOPA itself could serve as a substrate for NA synthesis in these terminals, increasing the total NA pool in the ipsilateral STR. In the contralateral STR, where all noradrenergic pathways were intact, inhibition of the NET was responsible for the increases in the NA content in the group receiving DES alone or in combination with L-DOPA. The STR, unlike SN, HIP and PFC, is one of brain structures with a very low content of NA; hence, the unexpectedly high increases in the NA concentration in the contralateral STR after chronic administration of DES alone or in combination with L-DOPA may be the mechanism to compensate for the deficiency of this neurotransmitter in the ipsilateral STR. Regarding the impact of DES and L-DOPA on the level of NA in the SN, only in the ipsilateral SN a significant treatment effect of L-DOPA was observed, consisting in reducing the NA level in this structure.

Considering the role of the antidepressant DES in modulating motor behavior, it should be borne in mind that following its acute intraperitoneal administration to rats, this drug, by inhibiting the NET, increases the extracellular NA concentrations in the LC and other brainstem nuclei (A1 and A2 group). Then, NA, by activating inhibitory somatodendritic α2-adrenergic autoreceptors located on noradrenergic neurons, reduces its release at synaptic terminals in target regions of the brain (Mateo et al. 1998; Fernández-Pastor et al. 2005). It seems that in our study a transient decline in NA release in target brain structures (presumably in the NAcc shell) following the first combined dose of DES + L-DOPA may be related to a parallel slight decline in the number of contralateral rotations observed in the second part of the 2-h measurement period. However, chronic treatment with DES leads to the desensitization of presynaptic α2-adrenergic autoreceptors (Linnér et al. 1999) regulating the local release of NA both in the LC and the target brain structures (Mateo et al. 2001), and consequently to an increase in the extracellular NA levels. Referring the above-presented data to our study, it seems that in rats chronically treated with the DES + L-DOPA combination, the increase in NA concentration in the ipsilateral STR (presumably in the NAcc shell) may contribute to an upward trend in the number of contralateral rotations. In contrast to the effect described above, chronic treatment with L-DOPA alone did not increase the striatal NA content, and consistently the level of contralateral rotations in this group was slightly lower than in that receiving the DES + L-DOPA combination. The presented interpretation of the role of noradrenergic transmission in modulating motor behavior is consistent with an earlier study by Barnum et al. (2012) who showed that the number of L-DOPA-induced contralateral rotations in rats with simultaneous degenerations of two neurotransmission systems, i.e. the nigrostriatal dopaminergic and noradrenergic routes (as in PD), was significantly lower than in those with degeneration of only the nigrostriatal dopaminergic pathway. These data are also in line with some other studies which showed that antiparkinsonian pro-motor activity of L-DOPA was diminished in rats with concomitant lesions of DA and NA system compared to those with only a lesion of DA system (Archer and Fredriksson 2006; Nishi et al. 1991; Ostock et al. 2014). Therefore, to address the problem of improving motor functions in PD, it is necessary to determine the degree of degeneration of noradrenergic projections and the possibility of modulating the NA transmission in the specific brain structures.

The increase in the number of contralateral rotations in rats treated chronically with the DES + L-DOPA combination in our study corresponds well with the study by Swerdlow and Koob (1989), who showed that intraventricular (ICV) infusion of NA to rats with prior depletion of whole brain catecholamines by ICV injection of 6-OHDA resulted in a much stronger locomotor response compared to that in the sham-lesioned control group. Also, some other studies showed that direct injection of NA into the NAcc elicited locomotor activation and increased open-field exploration in rats (Svensson and Ahlenius 1982, Vance and Blumberg 1983, Płaźnik et al. 1985). Hence, in our study, a much stronger increase in the number of contralateral rotations after the combined administration of DES + L-DOPA than after L-DOPA alone indicates a much more beneficial effect of modulating both dopaminergic and noradrenergic transmission on locomotor activity than just increasing dopaminergic transmission by L-DOPA. However, this conclusion cannot be unequivocally translated into the expression of AIMs, as rotational behavior is not an equivalent of AIMs, and the direct measurement of AIMs under chronic DES + L-DOPA treatment was not performed in the PD model used in this study. In fact, the role of noradrenergic compounds in modulating L-DOPA induced AIMs is unclear. The effects of NET inhibitors analyzed in the first part of the discussion seem to suggest worsening (Chotibut et al. 2014) or no changes in the intensity of the L-DOPA-induced dyskinesia (Conti et al. 2016a). Moreover, it was shown that the local administration of NA into the STR of L-DOPA-primed rats, which previously received 6-OHDA unilaterally into the MFB, induced AIMs, as did local administration of L-DOPA into this structure (Buck and Ferger 2009). Since DES, as a potent NA reuptake inhibitor, also has the ability to inhibit 5-HT reuptake, our study should also consider the role of SERT blockade in modulating the L-DOPA-induced AIMs. It is commonly accepted that under conditions of the loss of nigrostriatal dopaminergic neurons, serotonergic neurons are mainly responsible for the conversion of exogenous L-DOPA into DA as well as for its storage in the synaptic vesicles and release to the synaptic cleft (Arai et al. 1994, 1995; Tanaka et al. 1999; Kannari et al. 2006; Maeda et al. 2005). However, serotonergic neurons lack mechanisms regulating DA release, such as DA D2 autoreceptors and DAT; hence, the uncontrolled release of L-DOPA-derived DA (Tanaka et al. 1999; Carta et al. 2007) and fluctuations in its extracellular level may play a causative role in the appearance of dyskinesias (Carta and Bezard 2011; Mosharov et al. 2015). It is also well known that L-DOPA can be further converted into NA inside noradrenergic terminals. It is worth noting that the SN has much stronger serotonergic and noradrenergic innervation than the STR. In the used rat model of PD, the serotonergic pathways from the RN and noradrenergic from the LC do not seem to be damaged by 6-OHDA injected into the MFB as both 5-HT and NA levels in the ipsi- and contralateral SN were equally high at both sides, in opposite to the STR in which significant decrease in both 5-HT and NA was found in the ipsilateral STR compared to the contralateral STR. Previously, Navailles et al. (2011) showed by means of multisite microdialysis performed in unilaterally 6-OHDA-lesioned rats that repeated administration of L-DOPA (12 mg/kg) for 10 days significantly reduced 5-HT release in the ipsilateral SNr, while in the ipsilateral STR only a decreasing tendency in 5-HT release was found. In the present study, chronic treatment with DES alone or in combination with L-DOPA significantly increased the tissue content of 5-HT including also its extracellular pool and simultaneously decreased the tissue NA content in the ipsilateral SN. In the ipsilateral STR no changes in the 5-HT content, and only a slight increase in the NA level was observed. The rise in the extracellular 5-HT and the increased activation of 5-HT_1A_ autoreceptors in the RN and 5-HT_1B_ autoreceptors at the serotonergic terminals mainly in the ipsilateral SNr and to a lesser extent in the ipsilateral STR can modulate excessive release of L-DOPA-derived DA and, consequently, can alleviate motor complications such as dyskinesias. The analysis presented above suggests that the simultaneous inhibition of NET and SERT appropriate to the degree of damage of the noradrenergic and serotonergic projections, respectively, may be of key importance in modulating the action of L-DOPA on motor functions in the rat model of PD induced by 6-OHDA administration to the MFB. However, this assumption requires confirmation by further studies.

On the other hand, the use of either agonists or antagonists of the α2 noradrenergic receptors reduced AIMs in rodents (Lundblad et al. 2002, Dekundy et al. 2007, Rommelfanger and Weinshenker 2007, Buck et al. 2010, Wang et al. 2014, Ostock et al. 2015), non-human primates (Henry et al. 1999; Grondin et al. 2000) and in parkinsonian patients (Rascol et al. 2001). However, some of these compounds show ambiguous effects on therapeutic benefits evoked by L-DOPA. The classic α2 adrenoceptor agonist, clonidine, weakens AIMs but also reduces the antiparkinsonian motor effects of L-DOPA (Gomez-Mancilla and Bedard 1993; Dekundy et al. 2007; Ostock et al. 2015), while several classic α2 adrenoceptor antagonists, such as yohimbine, idazoxan or fipamezole, reduce AIMs in experimental and clinical studies (Lundblad et al. 2002; Dekundy et al. 2007; Buck et al. 2010; Grondin et al. 2000; Barnum et al. 2012; Rascol et al. 2001) without compromising the antiparkinsonian motor benefits of L-DOPA (Henry et al. 1999). Some of these drugs are also weak 5-HT_1A_ receptors agonists. The paradoxical convergence of the action of α2 adrenoceptor agonists and antagonists in attenuating L-DOPA-induced AIMs suggests the existence of highly complex mechanisms underlying these effects that require further elucidation. The α2 adrenoceptors exist both as α2-autoreceptors, located on the cell bodies of the noradrenergic neurons in the LC and at their terminals in the target brain structures receiving noradrenergic innervation, and as heteroreceptors located on other neurons within these structures. In the α2 receptor family, the α2A adrenoceptor subtype acts as an inhibitory autoreceptor modulating noradrenergic transmission, while the α2C adrenoceptor subtype acts as a heteroreceptor modulating other neurotransmitter systems in brain structures innervated by noradrenergic projections. The highest concentration of α2C adrenoceptors is found in the STR, but they are also present in the LC and SN. Thus, the effect of α2-adrenoceptor agonists and antagonists on L-DOPA-induced dyskinesias may be a function of their action via the α2A and α2C adrenoceptor subtypes. However, one limitation in investigating the mechanisms of drug action by the pharmacology of α2A and α2C is related to the lack of selective ligands for these receptors. Therefore, it is necessary to search for such compounds and carry out further research on their effectiveness in modulating L-DOPA-induced dyskinesias in animal models of PD, in order to introduce noradrenergic compounds into L-DOPA therapy in the future.

### Impact of DES and L-DOPA on the psychiatric symptoms

The changes in the tissue concentrations of individual monoamines in the limbic brain structures of rats chronically administered DES + L-DOPA in connection with the treatment of depression coexisting with motor symptom are much more difficult to interpret than the changes in the concentrations of these monoamines in the motor brain structures in relation to improvement of the characteristic motor deficits. Depression is characterized by both emotional and cognitive symptoms, and interestingly, cognitive symptoms include deficits in attention, working memory and episodic memory (Disner et al. 2011; Millan et al. 2012). Furthermore, these symptoms have been linked to functional abnormalities of the HIP and PFC (Pittenger and Duman 2008; Clark et al. 2009). It is traditionally accepted that disturbances in NA, 5-HT and DA transmissions, in the limbic and cortical structures, underlie major depression. Depressive symptoms occur in at least 50% of PD patients on average (Barone 2011; Lemke 2008); they appear in the presymptomatic (non-motor) phase of the disease and persist during the symptomatic stage. Overall, in the presymptomatic phase, the coexistence of neuropsychiatric symptoms is mainly attributed to dysfunctions of the noradrenergic and serotonergic systems; however, it seems that progressive degeneration of the mesocortical and mesolimbic dopaminergic pathways may play a more important role in the manifestation of these symptoms than previously thought.

L-DOPA is the most common and effective treatment for the motor symptoms of PD, but its contribution to improving mood-related symptoms is controversial. In some clinical studies a slight improvement in mood was shown after L-DOPA (Funkiewiez et al. 2006; Growdon et al. 1998; Witt et al 2006) while others have not confirmed the antidepressant effect of this drug (Marsh and Markham 1973; Kim et al. 2009). Moreover, in healthy human volunteers L-DOPA did not affect positive mood (Liggins et al. 2012), in contrast to psychostimulant drugs which reliably and potently improved it. The lack of positive effect of L-DOPA on mood may be associated with specificity of this drug which must be transformed to DA to exert its biological function. As previously demonstrated by Navailles et al. (2010) L-DOPA in a wide range of doses enhanced the extracellular DA level in the brain regions receiving abundant serotonergic innervation, i.e. STR, SN, HIP and PFC. The destruction of serotonergic projections by infusion of 5,7-dihydroxytryptamine significantly decreased the extracellular DA content, clearly indicating that L-DOPA is mainly converted to DA in the serotonergic terminals and released from them in the brain structures innervated by these projections (Navailles et al. 2010, 2011). Moreover, DA derived from L-DOPA in serotonergic terminals competes with 5-HT for storage in synaptic vesicles, displaces 5-HT to the cytoplasm and accelerates its turnover, ultimately reducing the concentration of 5-HT in specific brain structures. Thus, an increase in the 5-HIAA/5-HT metabolic index can be taken as a measure of the decrease in 5-HT concentration. In view of these data, treatment of PD patients with L-DOPA, by lowering the level of 5-HT in the limbic structures of the brain, in the face of progressive degeneration of the ascending serotonergic pathways from the raphe nuclei (RN), may lead to deterioration of their mental state. In our study, chronic administration of L-DOPA at a dose of 12 mg/kg significantly reduced 5-HT levels in the ipsi- and contralateral HIP, while DES, which, in addition to suppressing the NET, has the ability to inhibit the SERT, administered in combination with L-DOPA increased 5-HT content in this brain structure when compared to the effect of L-DOPA alone. The increase in the tissue level of 5-HT in the HIP of rats chronically administered DES + L-DOPA may be due to the inhibition of 5-HT reuptake by DES and consequently may mainly affect its extracellular pool. The proposed mechanism for increasing the total 5-HT pool in the HIP of these rats is in line with the observed reduction in catabolism of this neurotransmitter as measured by the 5-HIAA/5-HT metabolic rate. A decreased value of 5-HIAA/5-HT metabolic index was also found in the ipsi- and contralateral PFC, suggesting that the extracellular 5-HT pool should also be increased in this structure, but this metabolic change was not reflected by a significant increase in the tissue 5-HT concentration. The beneficial effects of DES administered in combination with L-DOPA leading to an increase in the extracellular 5-HT pool in the limbic structures of the rat brain, under conditions of the marked degeneration of the serotonergic pathways innervating these structures, suggest that modulation of serotonergic transmission in PD by the antidepressants may have some potential to relieve depressive symptoms accompanying this disease. These results also seem to suggest that starting the treatment of depression in the presymptomatic phase of PD should be more effective due to a less advanced degenerative process.

The PFC commands a range of “executive functions” engaged in the modulation of behavior, thought and affect to produce thoughtful and purposeful actions (Goldman-Rakic 1995; Gamo and Arnsten 2011). In the PFC, catecholamines exert a potent impact on the proper functioning of this structure in such a way that their too little or too high level impairs its function, while a moderate level is required for optimal function (Vijayraghavan et al. 2007; Gamo and Arnsten 2011). Consistently with this view, in our study, drastic declines in NA and DA levels in the ipsilateral PFC, as a result of the progressive degeneration of the ascending noradrenergic and dopaminergic pathways from the LC and VTA, respectively, could disrupt functioning of this brain structure. It is worth recalling that in the PFC, due to the low expression of DAT (Sesack et al. 1998) and abundance of the NET (Freed et al. 1995; Miner et al. 2003), an uptake of the extracellular DA is carried out exclusively by the NET (Carboni et al. 1990, 2006; Pozzi et al. 1994; Gresch et al. 1995; Morón et al. 2002; Yamamoto and Novotney 1998). Hence, in the PFC, under optimal conditions, the extracellular DA competes with extracellular NA for the NET, and both pools of these neurotransmitters are maintained at the levels that guarantee proper functioning of this structure.

In the PD model used in our study, due to the significant degeneration of the NET-expressing noradrenergic terminals in the ipsilateral PFC, chronic administration of DES alone or in combination with L-DOPA was unable to increase the tissue NA levels in this structure. On the other hand, despite the drastic loss of dopaminergic innervation in the ipsilateral PFC, L-DOPA administered alone or together with DES significantly increased DA levels in this structure, and interestingly, the increase in DA content after combined treatment was significantly higher than after L-DOPA alone. The latter data clearly indicate that in the DA-denervated PFC, as in the DA-denervated STR, L-DOPA was converted to DA in a much larger population of the preserved serotonergic terminals. In contrast to the lesioned ipsilateral PFC, in the intact contralateral PFC, DES administered alone increased the tissue NA content while the DES + L-DOPA combination induced slightly smaller increase in the NA level. Chronic treatment with DES desensitizes both the presynaptic α2-adrenergic receptors and the NET (Linnér et al. 1999; Benmansour et al. 2004), which are localized on NA neurons in the LC and at noradrenergic terminals in the target structures (Tejani-Butt 1992; Schroeter et al. 2000), ultimately leading to the increase in the extracellular NA level. Therefore, it is justified to assume that in our study, these mechanisms were responsible for the observed increases in the measured NA level, which occurred mainly in the extracellular pool of this neurotransmitter. In general, in the intact PFC, the tissue NA level is significantly lower than the tissue DA level. As for the tissue DA level in our study, in the intact contralateral PFC, both DES and L-DOPA administered separately induced significant increases in its content, while the combined administration of DES + L-DOPA resulted in a slightly weaker increase in the DA level. The above effects of the investigated drugs on DA levels in the contralateral PFC can be explained in several ways. Since the NET mainly controls the extracellular level of DA in the PFC, desensitization of this transporter in the contralateral PFC of rats chronically treated with DES alone resulted in a significant increase in the endogenous extracellular DA pool. Parallel with this increase in the extracellular DA concentration, there was also an increase in the NA content, meaning that the functional pools of these neurotransmitters were in balance, although shifted to slightly higher levels. L-DOPA as a source of exogenous DA, administered chronically alone, also significantly increased the tissue DA in the contralateral PFC. However, in the intact contralateral PFC of rats treated with L-DOPA, the increase in the DA level was not accompanied by the rise in NA content, which means that the balance of the functional pool of these neurotransmitters was shifted in favor of the extracellular DA. Finally, in the contralateral PFC of rats receiving the DES + L-DOPA combination, the increase in DA content was significant, albeit slightly lower, than in the groups receiving these drugs separately. Furthermore, in the contralateral PFC of rats treated with DES + L-DOPA parallel to the increase in the extracellular DA concentration, there was also an increase in the NA content, meaning that the functional pools of these neurotransmitters were in balance. This balance between the DA and NA content in the PFC is crucial for modulating the functions mediated by these neurotransmitters.

Recent studies indicate that within the PFC, noradrenergic α1 and α2 receptors exert unique modulatory actions across a range of distinct cognitive processes (Mao et al. 1999; Ramos et al. 2006; Gamo and Arnsten 2011). Specifically, high affinity post-synaptic α2 receptors, engaged in NA release at moderate rates and associated with mild arousal levels, promote working memory (Ramos et al. 2006). In contrast, lower affinity α1 receptors, engaged in NA release at higher rates and associated with high arousal conditions (e.g. stress), impair working memory (Mao et al. 1999; Berridge and Spencer 2016). Also, deficiency or excess of DA, via insufficient or excessive dopamine D1 receptor mediated signaling in the PFC, causes cognitive impairment (Zahrt et al. 1997, Goldman-Rakic et al. 2000; Vijayraghavan et al. 2007). In light of the above data, the combined administration of DES + L-DOPA may have a beneficial effect on the mental state of PD patients, due to modulation of both NA and DA level in the PFC, while treatment with L-DOPA alone may have a negative effect due to excess of L-DOPA-derived DA and the exacerbation of DA-mediated functions. The beneficial effect of the DES + L-DOPA combination postulated in our study based on the analysis of the tissue concentrations of monoamines and their metabolites in the PFC is consistent with clinical trials recommending DES as an effective drug in the treatment of depression associated with PD in patients who were maintained on DA replacement therapy (Devos et al. 2008; Seppi et al. 2011).

In addition to the PFC, the HIP is also well known as a brain region closely related with learning and memory, as well as emotions. NA deficiency in the HIP can affect neurogenesis and neuroplasticity and, consequently, can impair learning and memory skills. In our study, a unilateral administration of 6-OHDA into the MFB induced degeneration of noradrenergic and dopaminergic pathways innervating the ipsilateral HIP, leading to significant decreases in the NA and DA content in this structure, as previously described (Kamińska et al. 2017). In this PD model, neither DES nor L-DOPA administered alone or in combination altered the tissue NA levels in the ipsi- and contralateral HIP. However, L-DOPA significantly increased DA concentration both in the ipsi- and contralateral HIP. In general, HIP receives few dopaminergic projections from VTA and SNc and is characterized by a very low DA level, many times lower than in the PFC (Bischoff et al. 1979; Gasbarri et al. 1994; Verney et al. 1985). Moreover, in the HIP, where DAT expression is low (Mennicken et al. 1992; Borgkvist et al. 2012), a high level of the NET in the hippocampal noradrenergic terminals (Tejani-Butt 1992, Gehlert et al. 1995, Schroeter et al. 2000) is a major regulating factor of the extracellular DA level, similar to the PFC. Thus, in line with the above data, it seems clear that in the contralateral HIP of rats receiving DES + L-DOPA, NET desensitization could contribute to a significant increase in the extracellular pool of the L-DOPA-derived DA. Since the HIP receives relatively poor DA innervation, it is almost certain that on the ipsilateral side, the conversion of L-DOPA to DA took place exclusively at the serotonergic terminals. However, it cannot be ruled out that also in the contralateral HIP this conversion occurred in serotonergic terminals, because a significant decrease in 5-HT content was observed in the group of rats treated with L-DOPA alone.

In conclusion, a comparison of the modulating effect of DES and L-DOPA, administered alone or in combination, on the monoamine levels in the ipsilateral PFC and the ipsilateral HIP appears to indicate that in the late-stage disease, the efficacy of the combined therapy in improving patients’ mental health may be rather small. On the other hand, the same analysis performed for the intact contralateral PFC and the contralateral HIP suggests that in the case of less advanced degenerative process, such treatment may improve the balance between monoamines tested, which may have a positive effect on the mental state of PD patients. The above interpretation of the obtained results suggests that the factor determining the effectiveness of the combined therapy is the introduction of antidepressants with noradrenergic profile in the early stage of the disease, especially since these drugs also have neuroprotective properties (Coradazzi et al. 2016; Zhu et al. 2019).

## Supplementary Information

Below is the link to the electronic supplementary material.

Supplementary file1 (DOC 57 KB)

Supplementary file2 (DOC 59 KB)
