# Supplementary material for: Behavioral and neurochemical interactions of the tricyclic antidepressant drug desipramine with L-DOPA in 6-OHDA-lesioned rats. Implications for motor and psychiatric functions in Parkinson’s disease
Source: Psychopharmacology (Berl). 2022 Sep 30;239(11):3633–56. doi: 10.1007/s00213-022-06238-x (PMC9584871; doi:10.1007/s00213-022-06238-x)
Supplement: Supplementary file 1 — Supplementary file1 (DOC 57 KB) [file 213_2022_6238_MOESM1_ESM.doc]

Table 4. The effect of 21-day treatment with desipramine (DES; 10 mg/kg) and/or L-DOPA (12 mg/kg), alone and in combination, on the levels of dopamine metabolites in the striatum (STR) and substantia nigra (SN) of unilaterally 6-OHDA-lesioned rats.

| Brain structures | DOPAC/DA | | 3-MT/DA | | HVA/DA | |
| --- | --- | --- | --- | --- | --- | --- |
|  | Ipsilateral side  6-OHDA(L) | Contralateral side  Intact | Ipsilateral side  6-OHDA(L) | Contralateral side  Intact | Ipsilateral side  6-OHDA(L) | Contralateral side  Intact |
| ***Striatum***  L + veh | 71 ± 14 | 15.5 ± 2.3^ii^ | 20.1 ± 4.7 | 2.9 ± 0.1^ii^ | 23.5 ± 4 | 6.3 ± 0.5^ii^ |
| L + DES | 24 ± 3 | 9.2 ± 0.4^iii^ | 28.2 ± 4.0 | 2.6 ± 0.2^iii^ | 18.5 ± 2 | 4.9 ± 0.4^iii^ |
| L + L-DOPA | 147 ± 8 | 13.9 ± 1.2^iii^ | 9.2 ± 1.6 | 2.7 ± 0.2^ii^ | 146 ± 12^***,###^ | 9.3 ± 0.8^iii^ |
| L + DES + L-DOPA | 108 ± 23 | 13.0 ± 1.3^ii^ | 12.3 ± 2.4 | 2.0 ± 0.1^ii^ | 93 ± 18^***,###,∆∆∆^ | 8.8 ± 1^ii^ |
| *Effect of L-DOPA*  *Effect of DES*  *Interaction*  ***Substantia nigra***  L + veh | *F(1,35)=41.3, P<0.001*  *F91,35)=11.8, P<0.01*  *no*  19 ± 2 | *no*  *F(1,34)=6.1, P<0.05*  *no*  22.2 ± 3.2 | *F(1,35)=12.9, P<0.01*  *no*  *no*  3.7 ± 0.7 | *F(1,34)=5.1, P<0.05*  *F(1,34)=7.3, P<0.05*  *no*  4.1 ± 0.8 | *F(1,35) = 108.6, P<0.001*  *F(1,35) = 9.7, P<0.01*  *F(1,35) = 6.7, P<0.05*  5 ± 1 | *F(1,34)=28.0, P<0.001*  *no*  *no*  9.1 ± 1.1^ii^ |
| L + DES | 1 ± 0.3 | 13.5 ± 0.4^iii^ | 20.5 ± 3.8^***^ | 4.5 ± 0.4^ii^ | 11.1 ± 1.3 | 9.5 ± 0.7 |
| L + L-DOPA | 48 ± 8 | 41.1 ± 5 | 13.1 ± 3 | 3.8 ± 0.3^ii^ | 48.4 ± 6.7 | 31 ± 5^ii^ |
| L + DES + L-DOPA | 51 ± 12 | 43.0 ± 6.9 | 10.3 ± 1.9 | 3.6 ± 0.5^i^ | 52.2 ± 12 | 35 ± 7 |
| *Effect of L-DOPA*  *Effect of des*  *Interaction* | *F(1,37)=37.8, P<0.001*  *no*  *no* | *F(1,37)=34.3, P<0.001*  *no*  no | *no*  *F(1,37)=5.8, P<0.05*  *F(1,37)=11.5, P<0.01* | *no*  *no*  *no* | *F(1,37)=48.8, P<0.001*  *no*  no | *F(1,37)=40.6, P<0.001*  *no*  *no* |

One hour after administration of the last doses of the tested drugs, the rats were sacrificed, and the ipsi- and contralateral STR and SN tissue samples were separately dissected from their brains. The data are presented as the mean ± S.E.M., the number of rats per group was n = 8-12. Significance of differences in paired Student’s t-test ^i^P < 0.05, ^ii^P < 0.01, ^iii^P < 0.001 vs. ipsilateral side of respective group. Statistical significance of differences between all examined groups in the MAO-dependent-, COMT-dependent- and total DA catabolism assessed, respectively, as metabolic ratios of the intracellular concentrations of DA metabolite DOPAC to DA (DOPAC/DA), extracellular DA metabolite 3-MT to DA (3-MT/DA) or total DA metabolite HVA to DA (HVA/DA) in the STR and SN was calculated using a two-way ANOVA followed by the Newman-Keuls test when appropriate, ^***^P < 0.001 vs. L + veh-treated group, ^###^P < 0.001 vs. L + DES-treated group, ^∆∆∆^P < 0.001 vs. L + L-DOPA-treated group of the ipsilateral side.
