# Supplementary material for: Behavioral and neurochemical interactions of the tricyclic antidepressant drug desipramine with L-DOPA in 6-OHDA-lesioned rats. Implications for motor and psychiatric functions in Parkinson’s disease
Source: Psychopharmacology (Berl). 2022 Sep 30;239(11):3633–56. doi: 10.1007/s00213-022-06238-x (PMC9584871; doi:10.1007/s00213-022-06238-x)
Supplement: Supplementary file 2 — Supplementary file2 (DOC 59 KB) [file 213_2022_6238_MOESM2_ESM.doc]

Table 5. The effect of 21-day treatment with desipramine (DES; 10 mg/kg) and/or L-DOPA (12 mg/kg), alone and in combination, on the levels of dopamine metabolites in the prefrontal cortex (PFC) and hippocampus (HIP) of unilaterally 6-OHDA-lesioned rats.

| Brain structures | DOPAC/DA | | 3-MT/DA | | HVA/DA | |
| --- | --- | --- | --- | --- | --- | --- |
|  | Ipsilateral side  6-OHDA(L) | Contralateral side  Intact | Ipsilateral side  6-OHDA(L) | Contralateral side  Intact | Ipsilateral side  6-OHDA(L) | Contralateral side  Intact |
| ***Prefrontal cortex***  L + veh | 41 ± 5 | 20.2 ± 2.6^ii^ | 19.4 ± 2.1 | 2.7 ± 0.3^iii^ | 43 ± 5 | 10.9 ± 1^iii^ |
| L + DES | 20 ± 2 | 13.9 ± 0.7^ii^ | 40.2 ± 7.9 | 2.9 ± 0.2^iii^ | 35 ± 4 | 7.8± 1^iii^ |
| L + L-DOPA | 153 ± 27 | 25.8 ± 3.0^ii^ | 40.2 ± 17 | 4.3 ± 0.8^*^ | 230 ± 54 | 23.7 ± 4^ii^ |
| L + DES + L-DOPA | 115 ± 18 | 30.4 ± 3.2^iii^ | 7.9 ± 1.6 | 2.4 ± 0.2^∆,ii^ | 135 ± 21 | 28.8 ± 4^iii^ |
| *Effect of L-DOPA*  *Effect of DES*  *Interaction*  ***Hippocampus***  L + veh | *F(1,35)=47.1, P<0.001*  *no*  *no*  176 ± 24 | *F(1,35)=48.3, P<0.001*  *no*  *no*  74.1 ± 9.4^ii^ | *no*  *no*  *F(1,35)=7.6, P<0.01*  88.5 ± 19.4 | *no*  *no*  *F(1,35)=6.2, P<0.05*  38.3 ± 9.7 | *F(1,35) = 29.4, P<0.001*  *no*  *no*  89 ± 16 | *F(1,35)=48.3, P<0.001*  *no*  *no*  38.9 ± 7^i^ |
| L + DES | 99 ± 29 | 20.8 ± 1.2^***,i^ | 796 ± 213^**^ | 35.9 ± 7.4^ii^ | 237 ± 52^*^ | 18.7 ± 6^iii^ |
| L + L-DOPA | 176 ± 14 | 97.8 ± 4.6^###,ii^ | 12.4 ± 2.5^##^ | 5.3 ± 0.9^ii^ | 285 ± 46^**^ | 112 ± 8^,ii^ |
| L + DES + L-DOPA | 192 ± 52 | 78.8 ± 11^###^ | 39.6 ± 29^##^ | 9.1 ± 4.3 | 169 ± 23 | 79 ± 13^ii^ |
| *Effect of L-DOPA*  *Effect of DES*  *Interaction* | *no*  *no*  *no* | *F(1,35)=34.5, P<0.001*  *F(1,35)=27.1, P<0.001*  *F(1,35)=6.1, P<0.05* | *F(1,35)=9.2, P<0.01*  *F(1,35)=7.1, P<0.05*  *F(1,35)=6.1, P<0.05* | *F(1,35)=17.8, P<0.001*  *no*  *no* | *no*  *no*  *F(1,35)=10.5, P<0.01* | *F(1,35)=64.3, P<0.001*  *F(1,35)=10.1, P<0.01*  *no* |

One hour after administration of the last doses of the tested drugs, the rats were sacrificed, and the ipsi- and contralateral PFC and HIP tissue samples were separately dissected from their brains. The data are presented as the mean ± S.E.M., the number of rats per group was n = 8-12. Significance of differences in paired Student’s t-test ^i^P < 0.05, ^ii^P < 0.01, ^iii^P < 0.001 vs. ipsilateral side of respective group. Statistical significance of differences between all examined groups in the MAO-dependent-, COMT-dependent- and total DA catabolism assessed, respectively, as metabolic ratios of the intracellular concentrations of DA metabolite DOPAC to DA (DOPAC/DA), extracellular DA metabolite 3-MT to DA (3-MT/DA) or total DA metabolite HVA to DA (HVA/DA) in the PFC and HIP was calculated using a two-way ANOVA followed by the Newman-Keuls test when appropriate, ^*^P < 0.05, ^**^P < 0.01, ^***^P < 0.001 vs. L + veh-treated group, ^##^P < 0.01, ^###^P < 0.001 vs. L + DES-treated group, ^∆^P < 0.05 vs. L + L-DOPA-treated group of corresponding ipsi- or contralateral sides.
